# Gut-Expressed Vitellogenin Facilitates the Movement of a Plant Virus across the Midgut Wall in Its Insect Vector

**DOI:** 10.1128/mSystems.00581-21

**Published:** 2021-06-08

**Authors:** Ya-Zhou He, Yu-Meng Wang, Tian-Yan Yin, Wilmer J. Cuellar, Shu-Sheng Liu, Xiao-Wei Wang

**Affiliations:** a Ministry of Agriculture Key Laboratory of Molecular Biology of Crop Pathogens and insects, Institute of Insect Sciences, Zhejiang University, Hangzhou, China; b Virology Laboratory, International Center for Tropical Agriculture (CIAT), Cali, Colombia; University of California, San Diego

**Keywords:** vitellogenin, plant virus, insect vector, midgut barrier, whitefly

## Abstract

Many viral pathogens of global importance to plant and animal health are persistently transmitted by insect vectors. Midgut of insects forms the first major barrier that these viruses encounter during their entry into the vectors. However, the vector ligand(s) involved in the movement of plant viruses across the midgut barrier remains largely uncharacterized. Begomoviruses, many of which are disease agents of some major crops worldwide, are persistently transmitted by whiteflies (Bemisia tabaci). Here, in order to identify whitefly midgut proteins that interact with a devastating begomovirus, tomato yellow leaf curl virus (TYLCV), we performed midgut-specific TYLCV coat protein (CP) immunoprecipitation followed by high-throughput mass spectrometry proteomic analysis. We find that vitellogenin (Vg), a critical insect reproductive protein that has been considered to be synthesized by the fat body, is also synthesized by and interacts with TYLCV CP in the whitefly midgut. TYLCV appears to be internalized into midgut epithelial cells as a complex with Vg through endocytosis. Virus-containing vesicles then deliver the virus-Vg complexes to early endosomes for intracellular transport. Systematic silencing of Vg or midgut-specific immune blocking of Vg inhibited virus movement across the midgut wall and decreased viral acquisition and transmission by whitefly. Our findings show that a functional Vg protein is synthesized in the midgut of an insect and suggest a novel Vg mechanism that facilitates virus movement across the midgut barrier of its insect vector.

**IMPORTANCE** An essential step in the life cycle of many viruses is transmission to a new host by insect vectors, and one critical step in the transmission of persistently transmitted viruses is overcoming the midgut barrier to enter vectors and complete their cycle. Most viruses enter vector midgut epithelial cells via specific interaction between viral structural proteins and vector cell surface receptor complexes. Tomato yellow leaf curl virus (TYLCV) is persistently transmitted by the whitefly Bemisia tabaci between host plants. Here, we find that TYLCV coat protein interacts with vitellogenin (Vg) in the whitefly midgut. This interaction is required for the movement of the virus crossing the midgut wall and thus facilitates viral acquisition and transmission by whitefly. This study reveals a novel mechanism of virus overcoming the insect midgut barrier and provides new insights into the function of Vg beyond serving as nutrition for developing embryos in insects.

## INTRODUCTION

Many viruses that cause diseases in humans, animals, and plants are persistently transmitted by arthropod vectors, which notably encompass mosquitoes, ticks, whiteflies, leafhoppers, planthoppers, and aphids (1). After they are acquired from animal blood or plant sap by the arthropod, the viruses must first overcome the gut entry and dissemination barriers to get into the hemolymph or other tissues and finally into the salivary gland prior to being excreted and transmitted to new hosts (2). Previous studies have shown that many persistent viruses can be transmitted at higher efficiency when they are experimentally delivered into the hemocoel of the vector than when delivered via oral acquisition (3, 4) and that some viruses can even be transmitted by a nonvector when they are injected directly into the hemocoel of the insect (5, 6), illustrating the function of insect gut as a major barrier to transmission of persistent viruses. Passage of viruses through the gut barrier requires specific interactions between virus and vector components (2). Insect gut proteins that are involved in virus movement across the gut wall are considered important targets for virus transmission blockage (3, 7). However, with few exceptions, these vector molecules remain largely unknown for persistent plant viruses (2, 8–10).

*Begomovirus* (family *Geminiviridae*) is known as the largest genus of >400 species of plant viruses that are exclusively transmitted by whiteflies of the Bemisia tabaci cryptic species complex in a persistent manner (11–13). In the past 30 years, two species of the *B. tabaci* complex, provisionally named Middle East Asia Minor 1 (MEAM1, previously biotype B) and Mediterranean (MED, previously biotype Q), have invaded many regions of the world (11, 14). Along with the invasion by these two species of whiteflies, begomoviruses have emerged worldwide as serious constraints to the cultivation of a variety of economically important crops (15, 16). Tomato yellow leaf curl virus (TYLCV) is one of the most devastating begomoviruses and can be transmitted by a number of species of the whitefly complex such as MEAM1 and MED (17–19). After ingestion by the whitefly stylets from the phloem of infected plants, TYLCV passes along the food canal and reaches the esophagus after 10 min and the midgut after 40 min. TYLCV then moves across the midgut wall into the hemolymph after 90 min. From there, it further translocates into the primary salivary glands after 5.5 h. The virus is then egested with saliva into the plant phloem when whiteflies are feeding (20). TYLCV also invades the whitefly ovaries, from where it is vertically transmitted to the offspring (21). Complex interactions between the virus-encoded proteins and vector organs and proteins are involved in the circulation of begomoviruses in the whitefly body (18, 21, 22). TYLCV coat protein (CP) is considered the key viral component specifically interacting with whitefly proteins for virus transmission to occur (18, 23). Previous studies have provided evidence that TYLCV enters whitefly midgut epithelial cells through receptor-mediated, clathrin-dependent endocytosis (24) and that the early steps of endosome trafficking play an important role in the intracellular movement of the virus crossing the midgut wall (25). However, whitefly proteins mediating these processes remain largely unknown.

Vitellogenins (Vgs) are precursors of egg yolk that serve as the major source of nutrition for embryo development in almost all oviparous animals, including insects (26). Insect Vgs are considered to be synthesized in the female fat body, secreted into the hemolymph, and subsequently internalized by developing oocytes via Vg receptor (VgR)-mediated endocytosis (27). After synthesis in the fat body, Vg undergoes extensive structural alterations such as phosphorylation, lipidation, glycosylation, proteolytic cleavage, etc. (28, 29). The primary Vg precursors are usually cleaved into subunits at the conserved tetraresidue motif RXXR by subtilisin-like endoproteases (30, 31). This motif is mostly found conserved at the N terminus and is flanked by polyserine tracts (28). Cleavage of primary Vg at this motif results in an N-terminal small subunit and a C-terminal large subunit. In certain insects, this motif is also present at the C terminus or in the center, and the C-terminal large subunit is further cleaved into two medium-sized polypeptides (31–33).

In recent years, accumulating data have shown that the female fat body is not the only vitellogenic tissue of insects, as its synthesis also occurs in other female tissues as well as in males of some species (34–36). For example, Apis mellifera Vg is synthesized in the hypopharyngeal glands and the head fat bodies of functionally sterile worker bees, indicative of roles of Vg in the synthesis of brood food and social behavior (34). Leucophaea maderae Vg is expressed in the fat body of adult females and males. However, female- and male-produced Vgs are different in their polypeptide compositions, indicative of differences in Vg processing in the female and male fat body (35). In Laodelphax striatellus, abundant Vg protein is synthesized in the female hemocytes as well. However, the Vg is processed differently in the fat body and hemocytes, with both the N-terminal small subunit and the C-terminal rice stripe virus (RSV)-interacting large subunit existing stably in the hemocytes, whereas the large subunit is absent in the fat body. As a result, only the hemocyte-produced Vg binds to RSV *in vivo* and facilitates transmission of the virus (36). The tissue-specific expression and processing of insect Vgs revealed by these studies strongly indicate a multifunctional role of Vg in insects.

Previously, we demonstrated that the specific interaction between TYLCV CP and whitefly Vg is vital for TYLCV entry into the whitefly ovary. TYLCV binds to Vg in the hemolymph and then is transported into the oocyte as a complex with Vg (21). Here, we found that Vg protein is also synthesized in the whitefly midgut. Moreover, the gut-produced Vg interacts with TYLCV and facilitates the movement of TYLCV across the midgut wall for efficient transmission.

## RESULTS

### Isolation of whitefly proteins in the midgut that interact with TYLCV CP.

To identify whitefly midgut proteins that interact with TYLCV CP, total proteins were extracted from 2,000 midguts of viruliferous MEAM1 whiteflies and used for immunoprecipitation with a mouse anti-TYLCV CP monoclonal antibody or mouse preimmune sera (control sera). The immunoprecipitates were analyzed by shotgun ultraperformance liquid chromatography (UPLC)-tandem mass spectrometry (MS/MS), and the MS/MS spectra were searched against the peptide database of MEAM1 whitefly (http://www.whiteflygenomics.org) to identify TYLCV CP binding proteins in the midgut. Only proteins specifically precipitated by the anti-TYLCV CP antibody but not by the control sera and with at least two peptide-spectrum matches were considered credible candidates. Under these conditions, a total of 76 proteins were identified (see Data Set S1 in the supplemental material).

10.1128/mSystems.00581-21.10 DATA SET S1 Whitefly midgut proteins that specifically coimmunoprecipitated with anti-TYLCV CP antibody as identified by UPLC-MS/MS. Download Data Set S1, XLSX file, 0.04 MB. Copyright © 2021 He et al. 2021 He et al. https://creativecommons.org/licenses/by/4.0/ This content is distributed under the terms of the Creative Commons Attribution 4.0 International license.

To our surprise, a Vg protein (whitefly genome ID Bta07851) was isolated from midgut proteins of viruliferous whiteflies through the above approach (Data Set S1). The full-length cDNA of *Bta07851* is 6,624 bp in size and encodes a protein of 2,207 amino acid residues (Fig. S1A). Domain architecture analysis of the protein sequence showed the presence of the three Vg-specific functional domains: a vitellogenin N-terminal domain (VitN), a middle-region domain of unknown function (DUF1943), and a von Willebrand factor type D C-terminal domain (vWD) (Fig. S1A) (28, 31). Our previous studies have identified a MEAM1 whitefly Vg (GenBank accession no. GU332720.1) that interacts with TYLCV CP and facilitates the entry of TYLCV into the whitefly ovary for transovarial transmission (21, 37). A search of the MEAM1 whitefly genome database with *GU332720.1* showed that only *Bta07851* matched *GU332720.1* with a high score (12,470 bits), and all the other genes exhibited scores of <1,149 bits. The identity of the gene sequences between *Bta07851* and *GU332720.1* is 99.5%, although they are generated through different methods. Thus, *Bta07851* and *GU332720.1* are the same *Vg* gene. Since the interaction between TYLCV CP and Vg plays a critical role in virus overcoming the transovarial transmission barrier, the identification of Vg as a TYLCV CP-interacting protein in the midgut then leads us to test the possibility that the TYLCV CP-Vg interaction is also involved in virus passing through the midgut barrier in whiteflies.

10.1128/mSystems.00581-21.1 FIG S1 Vg subunit composition and Vg protein levels in the midgut and fat body of adult female whiteflies at two developmental stages. (A) Domain and subunit composition of Bta07851 (Vg). Domain composition is predicted using the Conserved Domain Database (https://www.ncbi.nlm.nih.gov/cdd). The amino acid sequences of domains are indicated by color: VitN (blue), DUF1943 (purple), and vWD (green). N-VitN, N-terminal region of VitN. C-VitN, C-terminal region of VitN. The span of Vg subunits is indicated by pairs of arrows. The conserved cleavage site is shown in black letters on a red background. An N-terminal signal peptide is predicted using the SignalP-5.0 Server (http://www.cbs.dtu.dk/services/SignalP/) and shown in black letters on a blue background. Peptides identified by UPLC-MS/MS following TYLCV CP antibody immunoprecipitation are shaded in yellow. (B) Vg monoclonal antibody specificity test. A single protein of 196 kDa was detected by the anti-Vg monoclonal antibody in total protein extract from adult female whiteflies. M, marker. (C) Detection of Vg protein in the midgut and fat body of adult female whiteflies using the anti-Vg monoclonal antibody. The molecular weight was indicated on the right. Download FIG S1, TIF file, 2.9 MB. Copyright © 2021 He et al. 2021 He et al. https://creativecommons.org/licenses/by/4.0/ This content is distributed under the terms of the Creative Commons Attribution 4.0 International license.

### Vg is expressed in the whitefly midgut.

**FIG 1:**
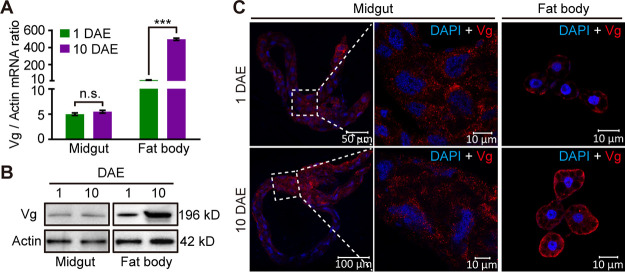
Vg is also expressed in the whitefly midgut. (A) Relative *Vg* mRNA levels in the midgut and fat body of adult female whiteflies at two developmental stages. Mean ± SEM from three independent experiments. n.s., not significant; ***, *P < *0.001 (independent-sample *t* test). (B) Vg protein levels in the midgut and fat body of adult female whiteflies at two developmental stages. The molecular weight was indicated on the right. (C) Immunofluorescence staining of Vg protein in the midgut and fat body of adult female whiteflies at two developmental stages. Vg was detected using a mouse anti-Vg monoclonal antibody and goat anti-mouse IgG labeled with DyLight 549 (red) secondary antibody. Cell nucleus was stained with DAPI (blue). Images are representative of three independent experiments with a total of 20 whiteflies analyzed for each time point.

To determine whether Bta07851 (here referred to as Vg for MEAM1 whitefly) is expressed in the whitefly midgut, we analyzed *Vg* transcript levels in the midgut of female MEAM1 whiteflies at different developmental stages using quantitative reverse transcription-PCR (qRT-PCR). As a control, the transcript levels of *Vg* in the female whitefly fat body were also monitored. *Vg* mRNA was constantly detected in the midguts of adult whiteflies at 1 and 10 days after eclosion (DAE). Notably, whereas the mRNA level of *Vg* in the fat body dramatically increased with whitefly development, it was stable in the midgut of adult whiteflies at 1 and 10 DAE (Fig. 1A).

Almost all insect primary Vg precursors are cleaved into subunits at the conserved RXXR motif by subtilisin-like endoproteases after synthesis (30–32). A detailed inspection of the full-length Vg protein sequence revealed the presence of one such motif: RSRR ending at amino acid 460. The predicted molecular weight of the full-length Vg protein was approximately 246 kDa. Cleavage at this site would result in an N-terminal small subunit with a predicted molecular weight of 49 kDa and a C-terminal large subunit of 196 kDa (Fig. S1A). Previous studies have shown that the whitefly vitellin (Vt) is an ∼380-kDa native molecule formed by two ∼190-kDa subunits (37, 38). These results further strengthened the cleavage of the primary Vg protein at this conserved motif.

A mouse anti-Vg monoclonal antibody has been developed using purified Vt extracted from ovaries of MEAM1 whiteflies as antigen. Since the purified Vt is composed of two ∼190-kDa subunits, this antibody recognizes the large subunit of whitefly Vg (37). Consistently, a single protein of 196 kDa was detected from whole-body extracts of MEAM1 females with this antibody (Fig. S1B). We then examined the presence of Vg protein in the midgut of whiteflies using Western blotting. A single protein of 196 kDa was detected in both the midgut and fat body extracts of 1- and 10-DAE females (Fig. 1B and Fig. S1C). We further visualized Vg protein using immunofluorescence assays (IFAs). Specific signals were clearly observed in the midgut and fat body of whiteflies at 1 and 10 DAE (Fig. 1C). Consistent with the mRNA levels, the quantity of Vg protein increased in the fat body with whitefly development but remained stable in the midgut (Fig. 1B and C).

### Vg is secreted into gut lumen where it binds to the microvillar membrane.

**FIG 2:**
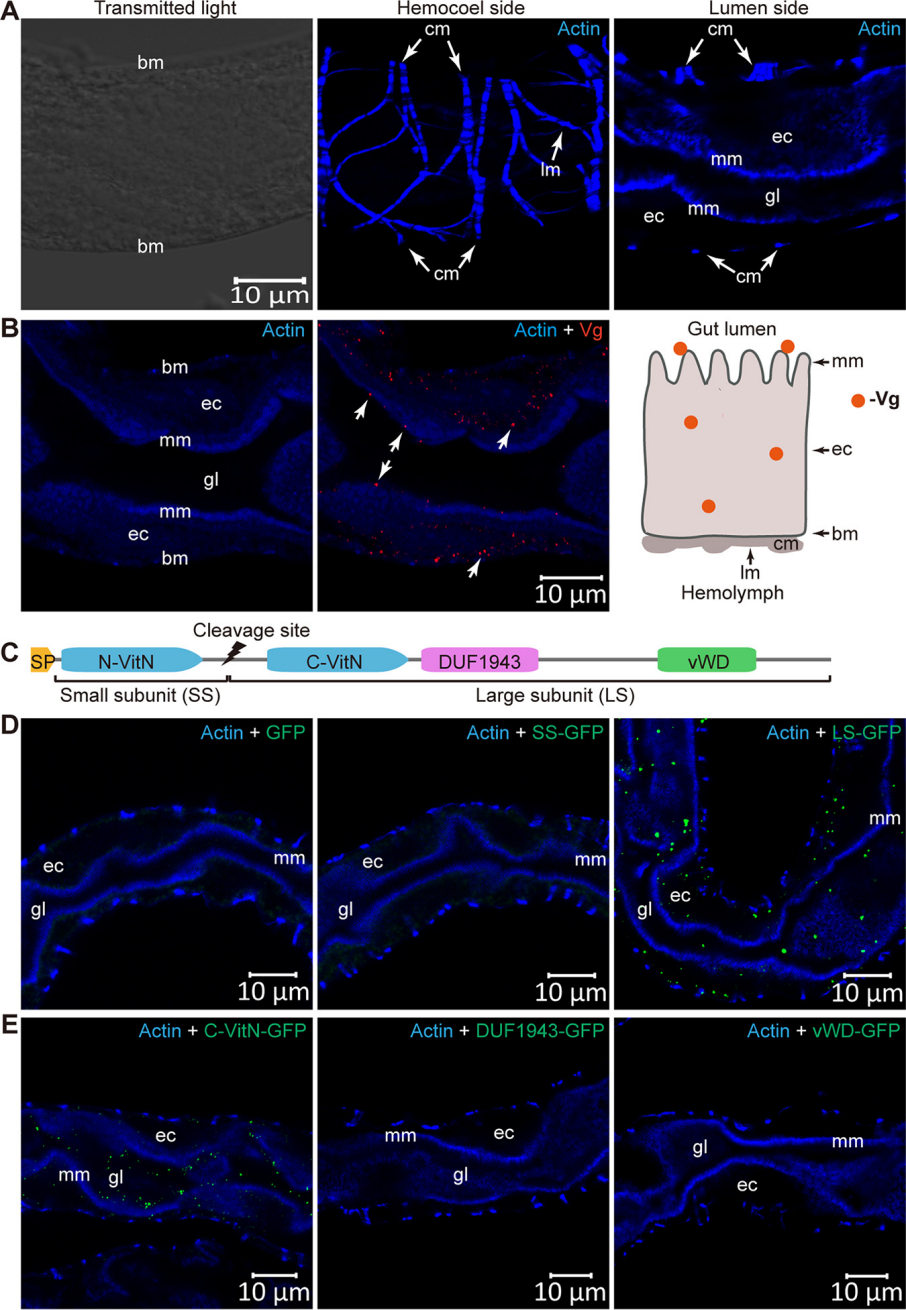
Vg is secreted into gut lumen where it binds to the microvillar membrane. (A) Alimentary canal structure of whitefly, composed of a single layer of epithelial cells, with microvillar membrane on the lumen side and basal membrane on the hemocoel side, covered with circular and longitudinal muscles. (B) Subcellular localization of Vg protein in midgut epithelial cells. The white arrow indicates the immunoreactive signal of Vg protein. Vg was detected using a mouse anti-Vg monoclonal antibody and goat anti-mouse IgG labeled with DyLight 549 (red) secondary antibody. Diagram of Vg distribution in midgut epithelium is shown on the right. (C) Diagram illustrating the subunit and domain composition of whitefly full-length Vg protein. SP, signal peptide. VitN, vitellogenin N-terminal domain. N-VitN, N-terminal region of VitN. C-VitN, C-terminal region of VitN. DUF1943, domain of unknown function. vWD, von Willebrand factor type D domain. (D) The large subunit of Vg binds to midgut microvillar membrane. (E) The C-VitN domain in the large subunit binds to midgut microvillar membrane. (D and E) Recombinant proteins were detected using a rabbit anti-GFP monoclonal antibody and goat anti-rabbit IgG labeled with DyLight 488 (green) secondary antibody. The actin-based microvillar membrane and muscle fibers were stained with DyLight 647 phalloidin (blue). bm, basal membrane; cm, circular muscle; lm, longitudinal muscle; gl, gut lumen; mm, microvillar membrane; ec, epithelial cell. Images are representative of three independent experiments with a total of 30 whiteflies analyzed for each treatment.

Insect Vgs are secreted proteins (28). To determine the distribution pattern of Vg in the midgut, we examined subcellular localization of Vg protein in midgut epithelial cells of female whiteflies. The alimentary canal of whitefly is composed of a single layer of epithelial cells, with microvillar membrane on the lumen side and basal membrane on the hemocoel side, covered with muscle fibers (Fig. 2A) (25). IFA showed the presence of Vg protein in the cytoplasm and at the lumen side of microvillar membrane (Fig. 2B), indicating that Vg is synthesized in the cytoplasm of midgut epithelial cells and secreted into gut lumen where it binds to the microvillar membrane. To test this hypothesis, the small subunit- and large subunit-green fluorescent protein (GFP) fusions were expressed in *Drosophila Schneider* 2 (S2) cells (Fig. S2A) and used for *in vivo* midgut binding assays (39). Whiteflies were fed with recombinant proteins for 4 h followed by a 6-h feeding on a sucrose solution to remove unbound proteins. IFA showed that the large subunit-GFP could be readily detected at the lumen side of microvillar membrane, whereas the small subunit-GFP or GFP alone was never detected (Fig. 2D). The Vg large subunit contains the C-terminal region of VitN (C-VitN), DUF1943, and vWD (Fig. 2C and Fig. S1A). To pinpoint the domains that mediate the interaction, the C-VitN-, DUF1943-, and vWD-GFP fusions were tested for binding to the midgut microvillar membrane. IFA revealed that C-VitN-GFP could be readily detected at the lumen side of microvillar membrane. In contrast, neither DUF1943-GFP nor vWD-GFP was detected in whitefly midguts (Fig. 2E). Specificity of the binding was further confirmed by feeding whiteflies with similar amounts of GFP and C-VitN-GFP, for which no binding was found for GFP alone (Fig. S2B). These results suggest that after synthesis in midgut cells, Vg is secreted into gut lumen where it binds to the microvillar membrane through C-VitN large subunit.

10.1128/mSystems.00581-21.2 FIG S2 Expression of Vg subunits and domains for *in vivo* midgut binding assays. (A) Expression of GFP, Vg small subunit-GFP, Vg large subunit-GFP, C-VitN-GFP, DUF1943-GFP, and vWD-GFP in *Drosophila Schneider* 2 (S2) cells. The pAc5.1/V5-His mock vector was used as control. The cells were visualized using a Zeiss LSM 780 confocal microscope with a 488-nm laser for GFP autofluorescence (green) excitation. (B) The C-VitN domain specifically binds to the whitefly midgut microvillar membrane. Whiteflies were fed with similar amounts of GFP or C-VitN-GFP for 4 h followed by a 6-h feeding on a sucrose solution to remove unbound proteins. Then, midguts were dissected for immunostaining using a rabbit anti-GFP monoclonal antibody and goat anti-rabbit IgG labeled with DyLight 488 (green) secondary antibody. The white arrow indicates the immunoreactive signal of the C-VitN-GFP. Western blotting of GFP and C-VitN-GFP serves as an input control (right). gl, gut lumen; mm, microvillar membrane; ec, epithelial cell; bm, basal membrane. Images are representative of three independent experiments with a total of 30 whiteflies analyzed for each treatment. Download FIG S2, TIF file, 6.8 MB. Copyright © 2021 He et al. 2021 He et al. https://creativecommons.org/licenses/by/4.0/ This content is distributed under the terms of the Creative Commons Attribution 4.0 International license.

### TYLCV interacts with Vg in the whitefly midgut.

**FIG 3:**
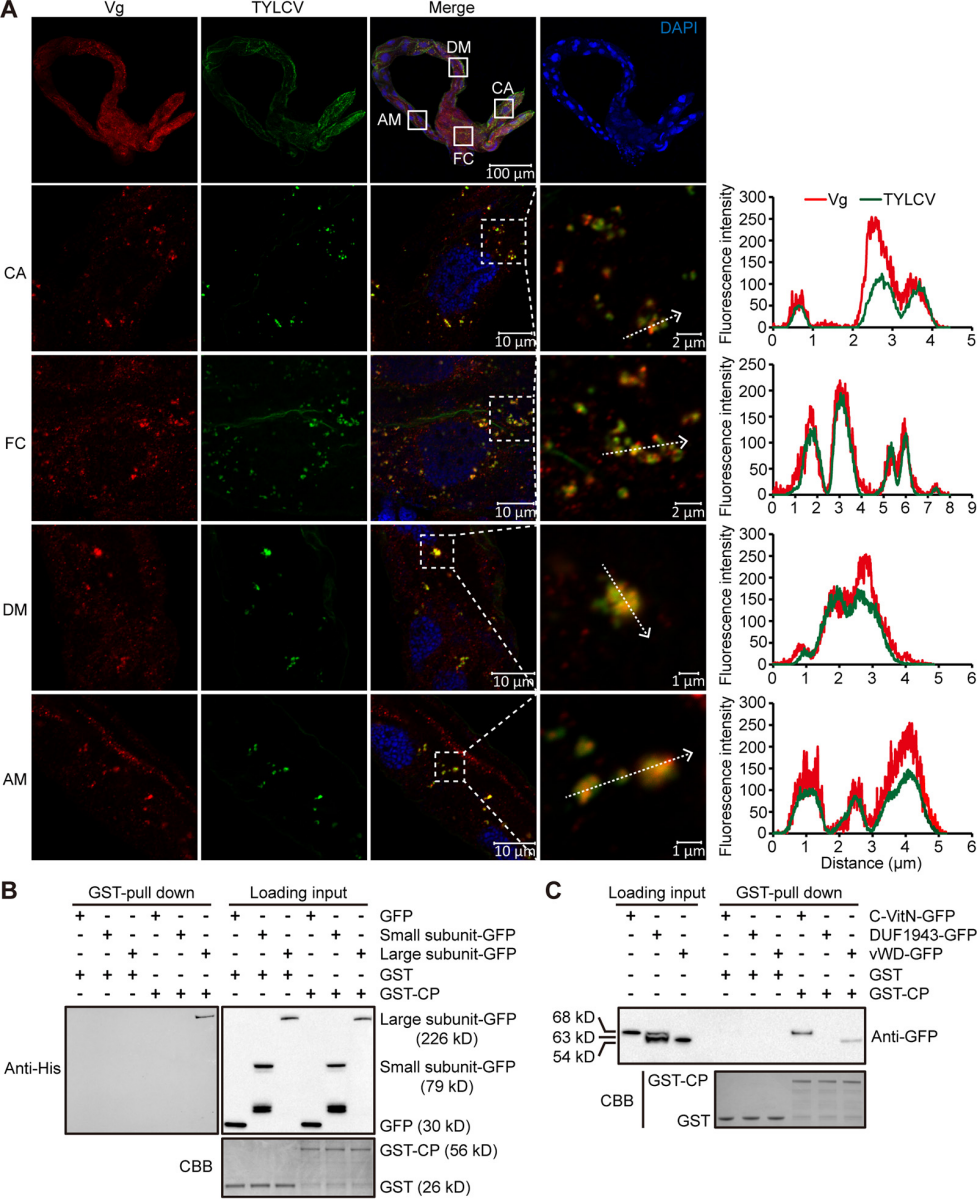
TYLCV interacts with Vg in the whitefly midgut. (A) Localization of TYLCV and Vg in different parts of the midgut from viruliferous female whitefly. Midguts of female whiteflies exposed to TYLCV-infected tomato plants for a 72-h acquisition access period (AAP) were dissected and used for immunofluorescence. Lower panels are the magnification of solid-line-boxed areas from gastric cecum (CA), filter chamber (FC), descending midgut (DM), and ascending midgut (AM) of the midgut in the first panel. Vg was detected using a mouse anti-Vg monoclonal antibody and goat anti-mouse IgG labeled with DyLight 549 (red) secondary antibody. TYLCV was detected using a rabbit anti-coat protein (CP) polyclonal antibody and goat anti-rabbit IgG labeled with DyLight 488 (green) secondary antibody. Cell nucleus was stained with DAPI (blue). Yellow color indicates the overlay of red and green. Analyses of overlapped fluorescence spectra from Vg and TYLCV in the boxed area were shown on the right of the images. The white dashed arrows mark the line scans and the direction used to create the fluorescence intensity profiles. Images are representative of three independent experiments with a total of 30 whiteflies analyzed. (B) Mapping of Vg subunits interacting with TYLCV CP using GST-pulldown assay. (C) Mapping of Vg domains interacting with TYLCV CP using GST-pulldown assay. Coomassie brilliant blue (CBB) staining of purified GST and GST-CP serves as a loading control.

To elucidate the relationship between TYLCV and Vg in the whitefly midgut, first, we examined the localization of TYLCV and Vg in the midgut of viruliferous whiteflies. The whitefly midgut consists of the gastric cecum, filter chamber, descending midgut, and ascending midgut (Fig. 3A). IFA showed that TYLCV colocalized with Vg in all these parts of the midgut (Fig. 3A), confirming the association of TYLCV with Vg in the whitefly midgut. Next, we investigated whether TYLCV CP binds to Vg and which subunits of Vg mediate the binding using a glutathione *S*-transferase (GST) pulldown assay. GST-fused TYLCV CP could bind to large subunit-GFP, but none bound to small subunit-GFP or GFP alone. Meanwhile, GST alone could not bind to the large subunit-GFP (Fig. 3B). We further examined which domains of the Vg large subunit mediate the binding and found that C-VitN and vWD could bind to GST-fused TYLCV CP, but none bound to GST alone (Fig. 3C). These results, together with the previous immunoprecipitation followed by UPLC-MS/MS analyses, demonstrate that TYLCV interacts with the large subunit of Vg in the whitefly midgut.

### TYLCV moves across midgut epithelial cells as a complex with Vg.

In order to understand the role of the interactions between TYLCV CP and Vg in virus movement across the midgut epithelial cells, first, we traced the time course movement of TYLCV in the midgut by IFA. The movement process could be classified into five phases. In phase I, TYLCV was detected only in the filter chamber of 23% of the tested midguts of whiteflies after a 1-h acquisition access period (AAP). In phase II, TYLCV was also detected in the gastric cecum and descending midgut of 40% of the tested midguts after a 1-h AAP and 60% of them after a 3-h AAP. In phase III, the virus was seen throughout the midgut in 44% of the tested midguts after a 6-h AAP. In phase II and phase III, the virus was bound to the microvillar membrane and not seen in the cytoplasm. In phase IV, some viral signals were seen in the cytoplasm of epithelial cells in 60% of the tested midguts after a 12-h AAP. In phase V, most viral signals were seen in the cytoplasm close to the basal membrane of epithelial cells in 50% of the tested midguts after a 24-h AAP and 93% after a 48-h AAP (Fig. S3 and Table S1). These observations indicate that after entering insect midgut lumen with plant sap, TYLCV first binds to the microvillar membrane and then invades the cytoplasm and moves to the basal membrane, where it can be released into the hemolymph for further spread.

10.1128/mSystems.00581-21.3 FIG S3 Time-course movement of TYLCV in the whitefly midgut. Midguts of female whiteflies feeding on TYLCV-infected tomato plants for AAPs of 1, 3, 6, 12, 24, and 48 h were dissected and prepared for immunofluorescence. After a 1-h AAP, virus signals were detected only in the filter chamber (A, phase I) or also detected in the gastric cecum and descending midgut (B, phase II). After a 3-h AAP, most of the midguts appeared as phase II, and after a 6-h APP, TYLCV was seen throughout the midgut (C, phase III). In phase II and phase III, the virus was bound to the microvillar membrane and not seen in the cytoplasm. After a 12-h AAP, some viral signals were seen in the cytoplasm of epithelial cells (D, phase IV). After a 24- and 48-h AAP, most viral signals were seen in the cytoplasm close to the basal membrane (E, phase V). TYLCV was detected using a mouse anti-CP monoclonal antibody and goat anti-mouse IgG labeled with DyLight 549 (red) secondary antibody. Cell nucleus was stained with DAPI (blue). Actin-based microvillar membrane and visceral muscles were stained with DyLight 647 phalloidin (green). Yellow arrow indicates viruses bound to the microvillar membrane. Reddish-purple arrow indicates viruses that have invaded the cytoplasm. gl, gut lumen; mm, microvillar membrane; vm, visceral muscles. Images are representative of a total of 30 midguts examined for each time point. Download FIG S3, TIF file, 9.8 MB. Copyright © 2021 He et al. 2021 He et al. https://creativecommons.org/licenses/by/4.0/ This content is distributed under the terms of the Creative Commons Attribution 4.0 International license.

10.1128/mSystems.00581-21.7 TABLE S1 Detection of TYLCV coat protein in the midguts of whiteflies by immunofluorescence microscopy at various times following the first access of whitefly to TYLCV-infected tomato plants. Download Table S1, PDF file, 0.06 MB. Copyright © 2021 He et al. 2021 He et al. https://creativecommons.org/licenses/by/4.0/ This content is distributed under the terms of the Creative Commons Attribution 4.0 International license.

**FIG 4:**
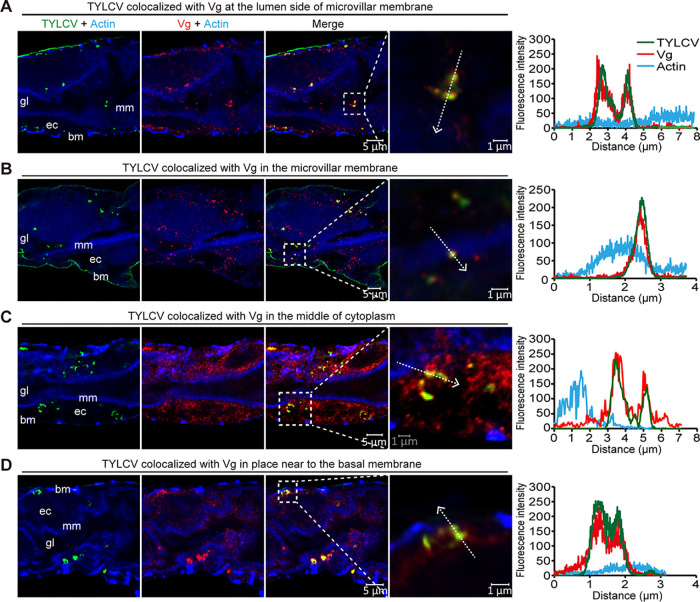
Subcellular localization of TYLCV and Vg in midgut epithelial cells. TYLCV colocalized with Vg at the lumen side of microvillar membrane (A), in the microvillar membrane (B), in the middle of cytoplasm (C), and in a place near the basal membrane (D). Midguts of female whiteflies exposed to TYLCV-infected plants for a 72-h AAP were dissected and used for immunofluorescence. TYLCV was detected using a rabbit anti-CP polyclonal antibody and goat anti-rabbit IgG labeled with DyLight 488 (green) secondary antibody. Vg was detected using a mouse anti-Vg monoclonal antibody and goat anti-mouse IgG labeled with DyLight 549 (red) secondary antibody. Yellow color indicates colocalization of red and green. Actin-based microvillar membrane and visceral muscles were stained with DyLight 647 phalloidin (blue). Analyses of fluorescence spectra from Vg, TYLCV, and actin in the boxed area were shown on the right of the images. The white dashed arrows mark the line scans and the direction used to create the fluorescence intensity profiles. Images are representatives of multiple experiments with multiple preparations. gl, gut lumen; mm, microvillar membrane; ec, epithelial cell; bm, basal membrane.

**FIG 5:**
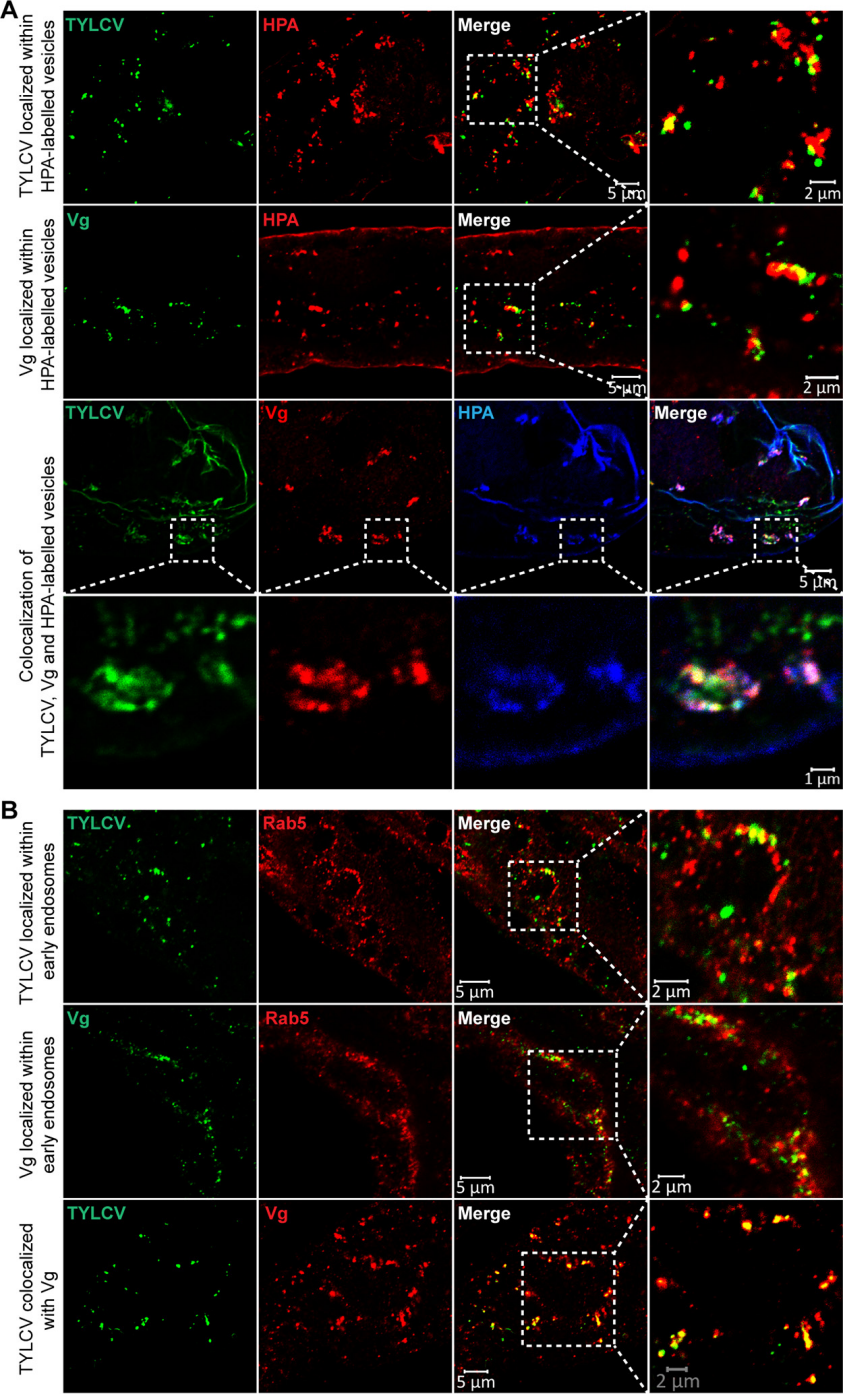
Colocalization of TYLCV and Vg with intracellular vesicles and early endosomes. Both TYLCV and Vg colocalized with lectin HPA-labeled vesicles (A) and Rab5-labeled early endosomes (B). Midguts of female whiteflies exposed to TYLCV-infected plants for a 72-h AAP were dissected and used for immunofluorescence. For colocalization of TYLCV and Vg with intracellular vesicles, TYLCV was detected using a rabbit anti-CP polyclonal antibody and goat anti-rabbit IgG labeled with DyLight 488 (green) secondary antibody. Intracellular vesicles were labeled using Alexa 647 lectin HPA. For colocalization of TYLCV and Vg with early endosomes, TYLCV was detected using a mouse anti-CP monoclonal antibody or rabbit anti-CP polyclonal antibody and the corresponding secondary antibody labeled with DyLight 488 (green). Early endosomes were detected using a rabbit anti-Rab5 polyclonal antibody and goat anti-rabbit IgG labeled with DyLight 549 (red) secondary antibody. Vg was detected using a mouse anti-Vg monoclonal antibody and goat anti-mouse IgG labeled with DyLight 488 (green) or DyLight 549 (red) secondary antibody. Yellow color indicates colocalization of red and green. Images are representative of multiple experiments with multiple preparations.

Next, we examined where the TYLCV-Vg interaction occurred during virus movement across the midgut epithelial cells. IFA showed that TYLCV colocalized with Vg at the lumen side of microvillar membrane (Fig. 4A), in the microvillar membrane (Fig. 4B), in the middle of cytoplasm (Fig. 4C), and in a place near the basal membrane (Fig. 4D), suggesting that TYLCV binds to Vg at the lumen side of microvillar membrane and moves first to the cytoplasm and then to the basal membrane as a complex with Vg. Previous studies have shown that vesicle trafficking is critical for the movement of TYLCV across the midgut wall in whiteflies. Particularly, almost all TYLCV colocalized with vesicles labeled with Helix pomatia agglutinin (HPA), an *N*-acetylgalactosamine binding lectin, within midgut epithelial cells (25). If the above hypothesis were correct, then Vg protein would also colocalize with HPA-labeled vesicles within midgut epithelial cells. Consequently, we examined the localization of TYLCV, Vg, and HPA-labeled vesicles in the midgut of viruliferous whiteflies and found that both TYLCV and Vg colocalized with HPA-labeled vesicles in the midgut (Fig. 5A). TYLCV was also found to be colocalized with early endosomes labeled with Rab5, an early endosome marker protein, in midgut epithelial cells (25). IFA further confirmed that both TYLCV and Vg colocalized with Rab5-labeled early endosomes within midgut epithelial cells (Fig. 5B). Taken together, these results clearly show that TYLCV moves across the midgut epithelial cells as a complex with Vg, suggesting an important role of the midgut-produced Vg in virus movement across the midgut wall.

### Silencing of Vg inhibits TYLCV movement across the midgut wall.

**FIG 6:**
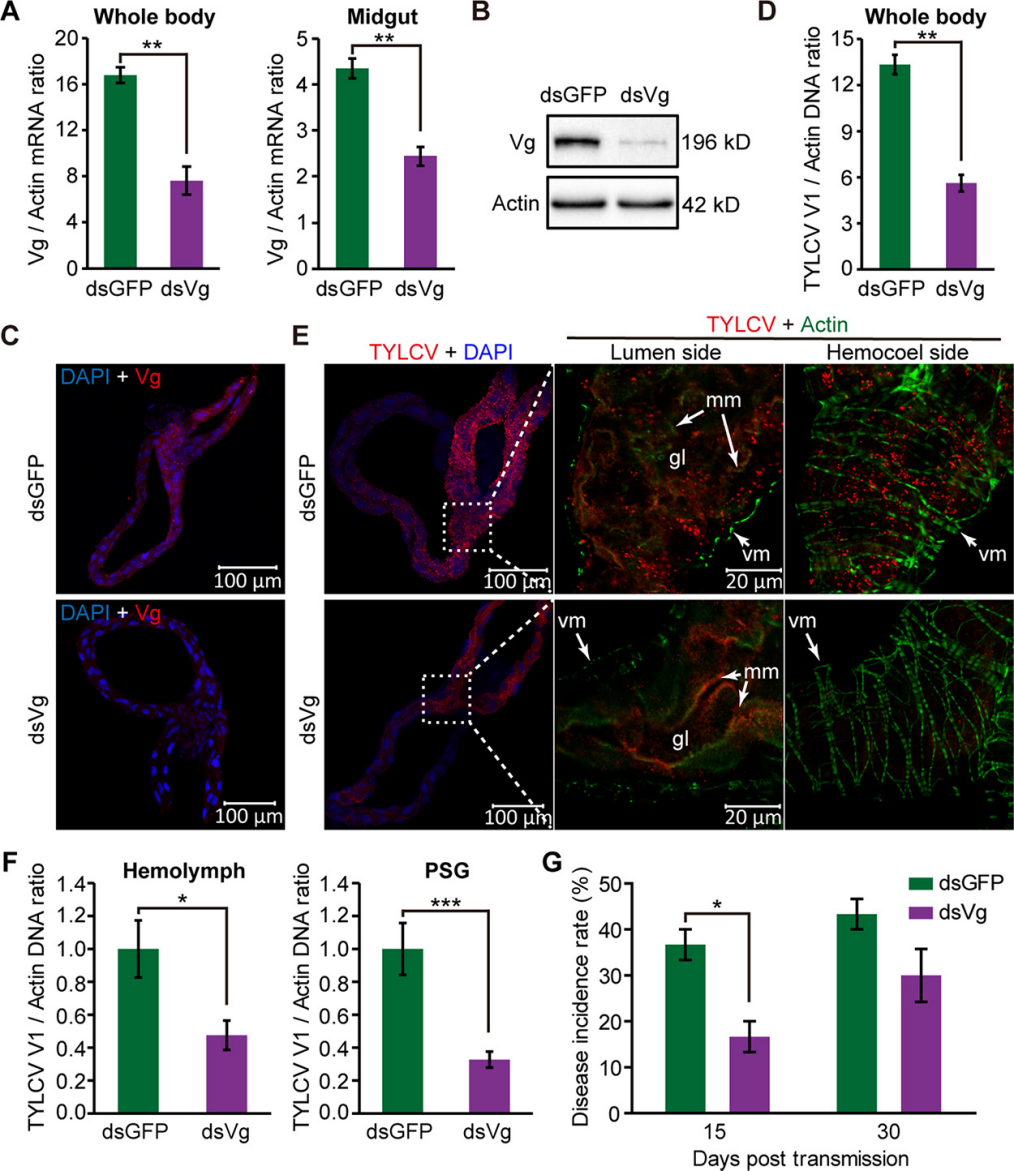
Silencing of Vg inhibits TYLCV movement across the midgut wall. (A) *Vg* mRNA levels in the whitefly whole body and midgut after feeding with dsRNAs. (B) Vg protein levels in whitefly whole body after feeding with dsRNAs. (C) Immunostaining of Vg protein in midguts of whiteflies after feeding with dsRNAs. Vg was detected using a mouse anti-Vg monoclonal antibody and goat anti-mouse IgG labeled with DyLight 549 (red) secondary antibody. Cell nucleus was stained with DAPI (blue). Images are representative of three independent experiments with a total of 20 whiteflies analyzed for each treatment. (D) TYLCV DNA levels in whitefly whole body after a 24-h AAP on TYLCV-infected tomato plants following dsRNA treatment. (E) Localization of TYLCV in midguts of dsRNA-treated whiteflies after a 24-h AAP on TYLCV-infected plants. TYLCV was detected using a mouse anti-CP monoclonal antibody and goat anti-mouse IgG labeled with DyLight 549 (red) secondary antibody. Cell nucleus was stained with DAPI (blue). Actin-based microvillar membrane and visceral muscles were stained with DyLight 647 phalloidin (green). gl, gut lumen; mm, microvillar membrane; vm, visceral muscles. Three independent experiments with a total of 60 whiteflies were analyzed for each treatment. For ds*GFP* treatment, images are representative of midguts at phase V. For ds*Vg* treatment, images are representative of midguts at phase IV. (F) TYLCV DNA levels in the hemolymph and primary salivary gland (PSG) of dsRNA-treated whiteflies after a 24-h AAP on TYLCV-infected plants. Mean ± SEM from 29 to 30 independent samples. One PSG or the hemolymph of one female whitefly was used as one sample for analyzing virus quantity. *, *P* < 0.05; ***, *P* < 0.001 (nonparametric Mann-Whitney *U* test). (G) The disease incidence rate of the tomato plants with TYLCV fed upon by dsRNA-treated whiteflies after a 24-h AAP on TYLCV-infected plants. (A, D, and G) Mean ± SEM from three independent experiments. *, *P* < 0.05; **, *P* < 0.01 (independent-sample *t* test).

To investigate the role of Vg in the movement of TYLCV across the midgut wall, we silenced the expression of Vg using RNA interference (RNAi). Compared with whiteflies fed with ds*GFP* (control), the *Vg* mRNA level decreased by 55% and 44% in whitefly whole body and midgut, respectively, in the group fed with ds*Vg* (Fig. 6A). The Vg protein levels in ds*Vg*-treated whitefly whole bodies and midguts also decreased as shown by Western blotting (Fig. 6B) and IFA (Fig. 6C). In order to compare the virus acquisition efficiencies, we put two groups of whiteflies on two opposite leaflets of a TYLCV-infected tomato plant (Fig. S4A). Quantitative PCR (qPCR) showed that, without treatment, quantities of virus acquired by whiteflies after a 24-h AAP on two opposite leaflets were similar (Fig. S4B). After ds*Vg* treatment, the abundance of TYLCV DNA in whitefly whole body is 58% lower than that in the ds*GFP*-treated insects (Fig. 6D). IFA showed that ds*Vg* treatment inhibited TYLCV movement across the midgut epithelial cells. More than half of the midguts were at phase V (51%) in ds*GFP*-treated whiteflies after a 24-h AAP. In contrast, the majority of the midguts in ds*Vg*-treated whiteflies were at phase II to IV (74%) (Fig. 6E and Table S2). We further analyzed the quantity of TYLCV that had moved across the midgut epithelial cells by quantifying virus abundance in the hemolymph and primary salivary glands (PSGs) of whiteflies after a 24-h AAP. Compared with the control, the abundance of TYLCV DNA in the hemolymph and PSGs of ds*Vg*-treated whiteflies decreased by 52% and 75%, respectively (Fig. 6F). In a virus transmission assay, 37% and 43% of the tomato plants in ds*GFP* treatment were symptomatic at 15 days and 30 days posttransmission, whereas only 17% and 30% of the plants in the ds*Vg* treatment were symptomatic at 15 days and 30 days posttransmission, respectively (Fig. 6G), indicating that silencing of Vg reduced virus transmission by whitefly.

10.1128/mSystems.00581-21.4 FIG S4 Schematic representation of the method used to compare virus acquisition efficiency of whiteflies. (A) Two groups of whiteflies were put on the opposite leaves of a TYLCV-infected tomato plant using leaf clip cages. (B) Similar virus amounts were acquired by whiteflies after a 24-h AAP on the two opposite leaves (left leaf and right leaf). Mean ± SEM from three independent experiments. Download FIG S4, TIF file, 2.8 MB. Copyright © 2021 He et al. 2021 He et al. https://creativecommons.org/licenses/by/4.0/ This content is distributed under the terms of the Creative Commons Attribution 4.0 International license.

10.1128/mSystems.00581-21.8 TABLE S2 Silencing of Vg or immune blocking of midgut Vg inhibited the movement of TYLCV crossing the midgut epithelial cells. Download Table S2, PDF file, 0.07 MB. Copyright © 2021 He et al. 2021 He et al. https://creativecommons.org/licenses/by/4.0/ This content is distributed under the terms of the Creative Commons Attribution 4.0 International license.

### Immune blocking of midgut Vg inhibits TYLCV movement across the midgut wall.

**FIG 7:**
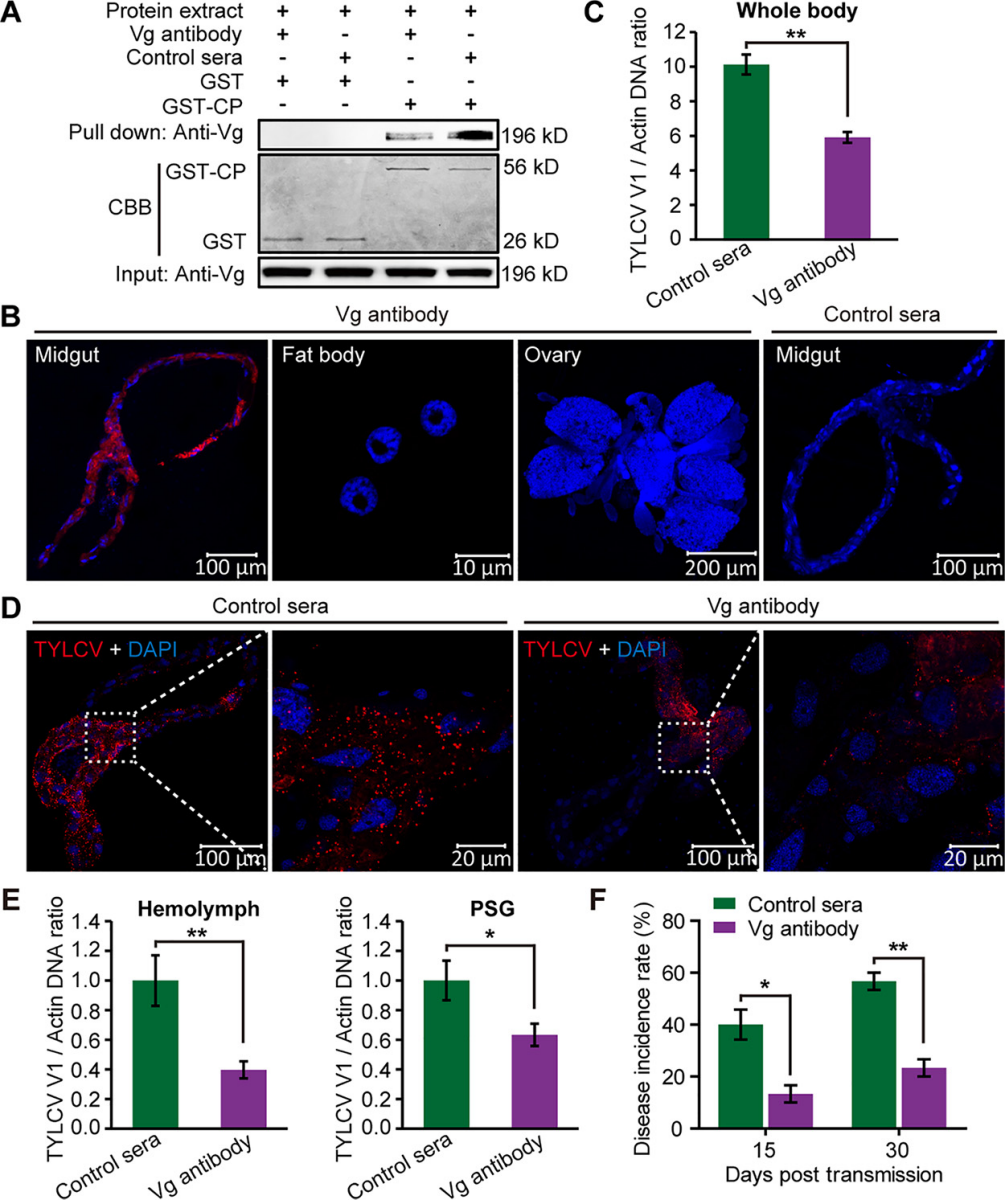
Immune blocking of midgut Vg inhibits TYLCV movement across the midgut wall. (A) Vg antibody interfered with the interaction between Vg and TYLCV CP. CBB staining of purified GST and GST-CP serves as a loading control. (B) Detection of ingested antibody in various tissues of whiteflies. Adult female whiteflies were fed with mouse anti-Vg monoclonal antibody or mouse preimmune serum (control serum) for 48 h via membrane feeding. Then, midguts, fat bodies, and ovaries were dissected and prepared for immunofluorescence. The Vg antibody and control serum were detected using a goat anti-mouse IgG labeled with DyLight 549 (red). (C) TYLCV DNA levels in antibody-treated whitefly whole bodies after a 24-h AAP on TYLCV-infected plants. (D) Localization of TYLCV in midguts of antibody-treated whiteflies after a 24-h AAP on TYLCV-infected tomato plants. TYLCV was detected using a mouse anti-CP monoclonal antibody and goat anti-mouse IgG labeled with DyLight 549 (red) secondary antibody. (B and D) Cell nucleus was stained with DAPI (blue). Images are representative of three independent experiments with a total of 30 whiteflies analyzed for each treatment. (E) TYLCV DNA levels in the hemolymph and PSG of Vg antibody- or control serum-treated whiteflies after a 24-h AAP on TYLCV-infected plants. Mean ± SEM from 29 to 30 independent samples. One PSG or the hemolymph of one female whitefly was used as one sample for analyzing virus quantity. *, *P* < 0.05; **, *P* < 0.01 (nonparametric Mann-Whitney *U* test). (F) The disease incidence rate of the tomato plants with TYLCV fed upon by antibody-treated whiteflies after a 24-h AAP on TYLCV-infected plants. (C and F) Mean ± SEM from three independent experiments. *, *P* < 0.05; **, *P* < 0.01 (independent-sample *t* test).

We further tested the function of Vg in the movement of TYLCV across the midgut wall by immune-blocking experiments. A GST-pulldown assay showed that Vg antibody interfered with Vg binding to GST-fused TYLCV CP (Fig. 7A). After oral ingestion of Vg antibody, the midgut, fat body, and ovary of female whitefly were dissected for Vg antibody detection. IFA showed abundant Vg antibodies in the midguts but no Vg antibody in the fat bodies or ovaries. Meanwhile, no specific signal was detected in the midguts of control serum-fed whiteflies (Fig. 7B). These results indicate that the ingested Vg antibody mainly located in the midgut of whitefly through binding with midgut Vg.

After a 24-h AAP on TYLCV-infected plants, the abundance of viral DNA in the whole body of Vg antibody-fed whiteflies was significantly lower than that in the control as revealed by qPCR (Fig. 7C). The movement of TYLCV in the midgut was also inhibited by Vg antibody treatment. IFA showed that the proportion of phase V midguts in Vg antibody-treated whiteflies (30%) was apparently lower than that in the control group (55%) after a 24-h AAP (Fig. 7D and Table S2). qPCR further revealed dramatically lower levels of TYLCV DNA in the hemolymph and PSG of Vg antibody-treated insects than in the control (Fig. 7E). In a virus transmission assay, the disease incidence rates of tomato plants at 15 days and 30 days posttransmission in the Vg antibody treatment were reduced by 27% and 35% compared with the control, respectively (Fig. 7F). Overall, the three sets of data indicate that, similarly to the effect of ds*Vg* treatment, immune blocking of midgut Vg inhibited virus movement across the midgut wall and reduced virus acquisition and transmission by whitefly.

### The role of VgR in TYLCV overcoming the midgut and ovary barriers in whiteflies.

Our previous results suggest that TYLCV binds to Vg at the lumen side of microvillar membrane and then enters midgut epithelial cells as a complex with Vg. The entry of Vg into the insect’s oocytes is mediated by VgR (27). A *VgR* gene from the MEAM1 whitefly has been cloned in a previous study (40). qRT-PCR and Western blotting showed that the *VgR* expression level in the whole body of nonviruliferous whitefly increased gradually from 1 to 10 DAE (Fig. S5A and B). In 10-DAE females, *VgR* was highly expressed in the ovary, while no VgR protein was detected in the midgut by Western blotting or IFA (Fig. S5C to E). In line with its role in mediating the uptake of Vg into oocytes, VgR was found to be colocalized with Vg in the space between follicular cells of the whitefly ovariole (Fig. S5F). To test whether VgR is involved in TYLCV overcoming the midgut and ovary barriers in whitefly, we silenced its expression using RNAi (Fig. S5G) and then analyzed the quantity of TYLCV in various tissues of whiteflies after a 24-h AAP. The abundances of TYLCV DNA in the whitefly whole body, hemolymph, and PSG were comparable between ds*VgR* and ds*GFP* treatments (Fig. S5H and I). In contrast, the abundance was significantly lower in the ovaries of ds*VgR*-treated whiteflies than in the control (Fig. S5I). These results demonstrate that VgR is synthesized specifically in the whitefly ovary and required for TYLCV to overcome the ovary barrier. The entry of TYLCV-Vg complexes into midgut epithelial cells is likely mediated by some other midgut-specific receptors.

10.1128/mSystems.00581-21.5 FIG S5 VgR is synthesized specifically in the ovary and required for TYLCV to overcome the ovary barrier. (A) Relative *VgR* mRNA levels in the whole bodies of adult female whiteflies at different developmental stages. (B) VgR protein levels in the whole bodies of adult female whiteflies at 1 and 10 days after eclosion. The molecular weight was indicated on the right. (C) Relative *VgR* mRNA levels in the midgut, fat body, and ovary of adult female whiteflies at 10 days after eclosion. (D) VgR protein levels in the midgut and fat body of adult female whiteflies at 10 days after eclosion. (E) Immunofluorescence staining of VgR protein in the midguts of adult female whiteflies at 10 days after eclosion. The actin-based microvillar membrane and muscle fibers were stained with DyLight 647 phalloidin (red). (F) Immunofluorescence staining of Vg and VgR proteins in the ovaries of adult female whiteflies at 10 days after eclosion. Vg was detected using a mouse anti-Vg monoclonal antibody and goat anti-mouse IgG labeled with DyLight 549 (red) secondary antibody. FC, follicular cells; O, oocyte. (E and F) VgR was detected using a rabbit anti-VgR polyclonal antibody and goat anti-rabbit IgG labeled with DyLight 488 (green) secondary antibody. Cell nucleus was stained with DAPI (blue). Images are representatives of multiple experiments with multiple preparations. (G) *VgR* mRNA levels in the whitefly whole body and midgut after feeding with dsRNAs. (H) TYLCV DNA levels in the whitefly whole body after a 24-h AAP on TYLCV-infected tomato plants following dsRNA treatment. (A, C, G, and H) Mean ± SEM from three independent experiments. n.s., not significant; ***, *P* < 0.001 (independent-sample *t* test). (I) TYLCV DNA levels in the hemolymph, primary salivary gland (PSG), and ovary of dsRNA-treated whiteflies after a 24-h AAP on TYLCV-infected plants. Mean ± SEM from 29 to 30 independent samples. One PSG, one ovary, or the hemolymph of one female whitefly was used as one sample for analyzing virus quantity. n.s., not significant; *, *P* < 0.05 (nonparametric Mann-Whitney *U* test). Download FIG S5, TIF file, 5.1 MB. Copyright © 2021 He et al. 2021 He et al. https://creativecommons.org/licenses/by/4.0/ This content is distributed under the terms of the Creative Commons Attribution 4.0 International license.

### Vg is synthesized in the midgut of male whiteflies and involved in TYLCV transmission.

**FIG 8:**
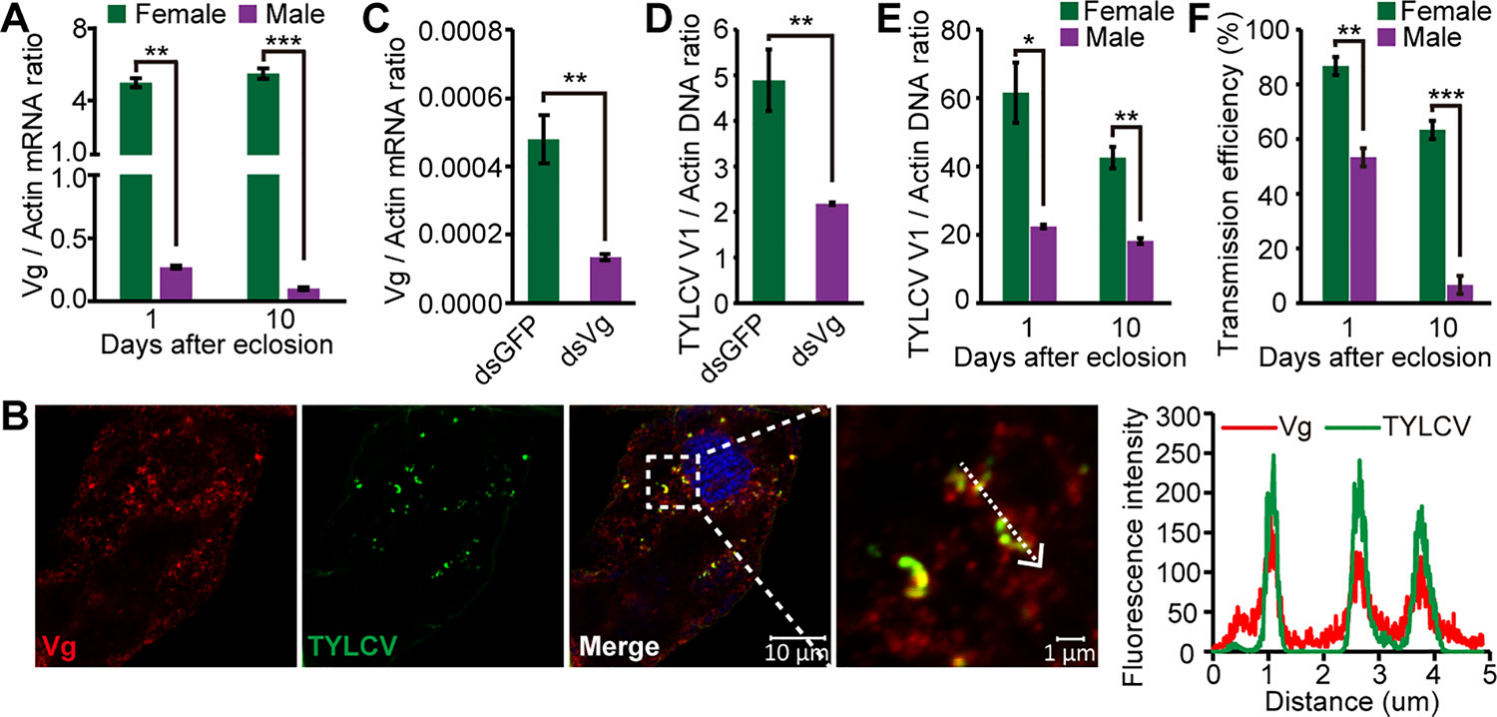
The role of Vg in TYLCV transmission by male whiteflies. (A) *Vg* mRNA levels in the midgut of adult female and male whiteflies at two developmental stages. (B) Localization of Vg and TYLCV in midgut epithelial cells of viruliferous male whiteflies. Vg was detected using a mouse anti-Vg monoclonal antibody and goat anti-mouse IgG labeled with DyLight 549 (red) secondary antibody. TYLCV was detected using a rabbit anti-CP polyclonal antibody and goat anti-rabbit IgG labeled with DyLight 488 (green) secondary antibody. Cell nucleus was stained with DAPI (blue). Yellow color indicates colocalization of red and green. Analysis of overlapped fluorescence spectra from Vg and TYLCV in the boxed area was shown on the right of the image. The white dashed arrow marks the line scans and the direction used to create the fluorescence intensity profiles. Images are representative of multiple experiments with multiple preparations. (C) *Vg* mRNA levels in male whitefly whole bodies after feeding with dsRNAs. (D) TYLCV DNA levels in whole body of dsRNA-treated males after a 24-h AAP on TYLCV-infected plants. (E) TYLCV DNA levels in whole body of female and male whiteflies at two developmental stages after a 48-h AAP on TYLCV-infected plants. (F) The inoculation capability of female and male adults at two developmental stages after a 48-h AAP on TYLCV-infected plants. For each combination, 10 plants per replicate and three replicates were conducted to examine the transmission efficiency. (A and C to F) Mean ± SEM from three independent experiments. *, *P* < 0.05; **, *P* < 0.01; ***, *P* < 0.001 (independent-sample *t* test).

Several studies have reported that insect Vg is also synthesized in males of some species (34–36). We thus investigated whether Vg is also expressed in the midgut of male whiteflies. qRT-PCR showed that *Vg* mRNA was produced in the midguts of MEAM1 males, with dramatically lower levels than that of the females (Fig. 8A). IFA further revealed the presence of Vg protein and the colocalization of TYLCV and Vg in the midgut epithelial cells of male whiteflies (Fig. 8B). Next, we silenced the expression of Vg in males using RNAi, and the *Vg* mRNA level in whole body was reduced by 72% in ds*Vg*-treated males compared with the control (Fig. 8C). The virus quantity in ds*Vg*-treated male whole body was significantly lower than that of the control after a 24-h AAP (Fig. 8D), suggesting that Vg also facilitates the movement of TYLCV across the midgut wall in male whiteflies.

Given the variable expression levels of *Vg* in the midgut of female and male whiteflies (Fig. 8A), we further compared the viral acquisition and transmission efficiencies between the two sexes. The viral acquisition and transmission efficiency was positively correlated with *Vg* expression levels in the midgut of female and male whiteflies (Fig. 8E and F). Therefore, the *Vg* expression level in the midgut may explain, at least in part, the differential acquisition and transmission efficiency of TYLCV by the two sexes.

### Functional conservation of the TYLCV CP-Vg interaction among different species of the *B. tabaci* complex.

**FIG 9:**
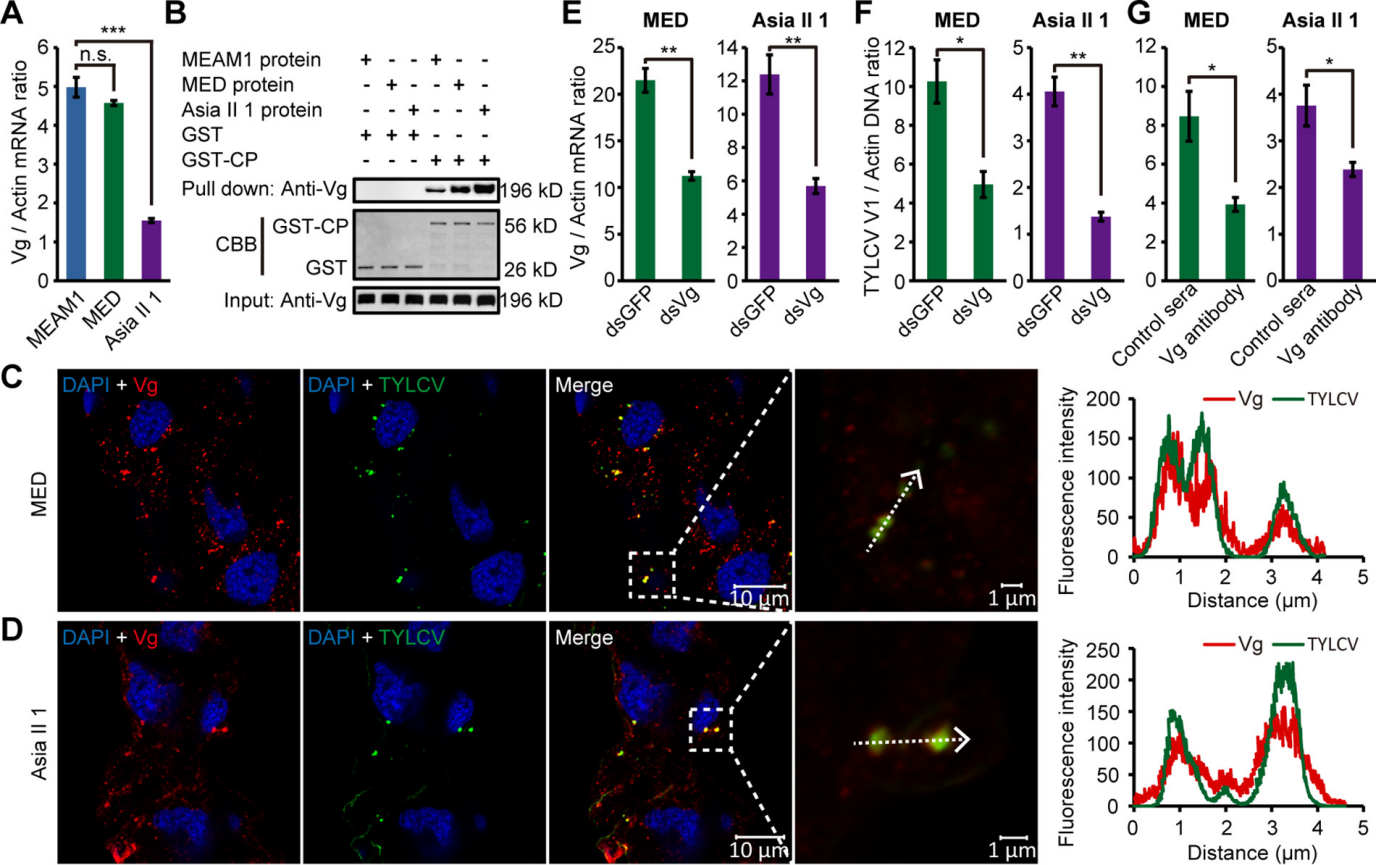
Functional conservation of the TYLCV CP-Vg interaction among different species of the *B. tabaci* complex. (A) *Vg* mRNA levels in the midgut of different whitefly species. (B) Vgs from three whitely species were all coeluted with GST-fused TYLCV CP but not with GST alone. CBB staining of purified GST and GST-CP serves as a loading control. (C and D) Localization of Vg and TYLCV in midgut epithelial cells of viruliferous whiteflies of MED (C) and Asia II 1 (D) species. Vg was detected using a mouse anti-Vg monoclonal antibody and goat anti-mouse IgG labeled with DyLight 549 (red) secondary antibody. TYLCV was detected using a rabbit anti-CP polyclonal antibody and goat anti-rabbit IgG labeled with DyLight 488 (green) secondary antibody. Cell nucleus was stained with DAPI (blue). Yellow color indicates the overlay of red and green. Analyses of overlapped fluorescence spectra from Vg and TYLCV in the boxed area were shown on the right of the images. The white dashed arrows mark the line scans and the direction used to create the fluorescence intensity profiles. Images are representative of multiple experiments with multiple preparations. (E) *Vg* mRNA levels in the whitefly whole body after feeding with dsRNAs. (F) TYLCV DNA levels in the whitefly whole body after a 24-h AAP on TYLCV-infected plants following dsRNA treatment. (G) TYLCV DNA levels in the whitefly whole body after a 24-h AAP on TYLCV-infected plants following antibody treatment. (A and E to G) Mean ± SEM from three independent experiments. n.s., not significant; *, *P* < 0.05; **, *P* < 0.01; ***, *P* < 0.001 (independent-sample *t* test).

Previous studies have shown that TYLCV is also efficiently acquired by the MED and Asia II 1 cryptic species of the *B. tabaci* complex (17); we thus investigated whether the TYLCV CP-Vg interaction also occurs in the midgut of these two whitefly species. The *Vg* genes of MED and Asia II 1 species have been cloned and sequenced in a previous study, resulting in fragments of 6,552 bp in MED (GU332722.1) and 6,549 bp in Asia II 1 (GU332721.1) (41). qRT-PCR revealed that both MED and Asia II 1 midguts produced *Vg* mRNA, with a level similar to that of MEAM1 in MED but a lower level in Asia II 1 (Fig. 9A). Aligning of the amino acid sequences of Vgs from MEAM1, MED, and Asia II 1 revealed high level of identities between these proteins, indicating evolutionarily conserved properties of Vgs in different whitefly species. Western blotting confirmed the recognition of Vgs from these three whitefly species by our anti-Vg antibody (Fig. 9B). GST-pulldown assay revealed that Vg proteins from MEAM1, MED, and Asia II 1 all coeluted with GST-fused TYLCV CP but not with GST alone (Fig. 9B). IFA further showed colocalization of TYLCV and Vg in the midgut epithelial cells of MED and Asia II 1 whiteflies (Fig. 9C and D). We then silenced the expression of Vg in MED and Asia II 1 whiteflies using RNAi. Compared with the control group, the *Vg* mRNA levels in the whole body decreased by 48% and 54% in ds*Vg*-treated MED and Asia II 1 whiteflies, respectively (Fig. 9E). After a 24-h AAP, the TYLCV abundance was decreased in both the ds*Vg*-treated MED and Asia II 1 whiteflies compared with the control (Fig. 9F). Moreover, immune blocking of midgut Vg also markedly reduced TYLCV quantity in whiteflies after a 24-h AAP (Fig. 9G). Therefore, the role of the TYLCV CP-Vg interaction in facilitating the movement of TYLCV across the midgut wall is conserved among different species of the *B. tabaci* complex.

## DISCUSSION

The gut wall of arthropod vectors serves as an important barrier for virus entry into vectors (7, 8). Most viruses enter cells via specific interaction between viral structural proteins and cell surface receptor complexes (42). Many animal virus receptors in the vector gut have been reported (7). For example, Aedes aegypti C-type lectin is induced in the midguts upon West Nile virus (WNV) infection and was found to be an interacting partner for WNV in the mosquitoes. Furthermore, CD45-like protein phosphatase of mosquitoes was shown to recruit C-type lectin to enable WNV attachment and entry into cells (43). Mercado-Curiel et al. identified two proteins with molecular masses of 80 (R80) and 67 (R67) kDa as receptors for dengue virus in A. aegypti midgut and Aedes albopictus cells, and specific antibodies generated against R80 or R67 inhibited cell binding and dengue virus infection (44). For plant viruses, only a few such proteins have been identified. Linz et al. reported *in vitro* evidence for the membrane alanyl aminopeptidase N of *Acyrthosiphon pisum* acting as a gut receptor of the pea enation mosaic virus (9). Recently, sugar transporter 6 has been identified as responsible for the entry of RSV into midgut epithelial cells of *L. striatellus* (10). Here, we identified Vg as an important vector protein that interacts with TYLCV in the midgut and facilitates TYLCV movement across the midgut wall in whiteflies. These findings improve our understanding of plant virus-insect vector interactions.

The movement of TYLCV across the midgut wall is a complicated multistep process, including attachment of virus to the microvillar membrane, entry of virus into epithelial cells, intracellular transport of virus to the basal membrane, and release of virus into the hemolymph (25). Multiple proteins may be required for each step, and different proteins may be involved in different steps. The entry of TYLCV into midgut cells depends on receptor-mediated, clathrin-dependent endocytosis (24). Intracellular transport of TYLCV relies on the early steps of endosome trafficking and Snx12-induced tubular vesicles (25). Our results showed that TYLCV moves across the midgut epithelial cells as a complex with Vg, starting at the lumen side of microvillar membrane, passing through the cytoplasm, and ending in places close to the basal membrane, suggesting that the Vg protein may be involved in the membrane attachment, cell entry, and intracellular transport of the virus in the whitefly midgut. After synthesis in the fat body, Vgs are secreted into the hemolymph and then absorbed into growing oocytes via VgR-mediated endocytosis (45). In the whitefly midgut, Vg is secreted into the gut lumen, where it binds to the microvillar membrane. In this case, the entry of Vg or Vg-TYLCV complex into epithelial cells should rely on some membrane-bound receptors. Functional analysis of an established whitefly *VgR* gene showed that this VgR is ovary-specifically expressed and only required for TYLCV entry into whitefly ovary. Some midgut-specific receptors may mediate the internalization of the Vg-TYLCV complex, and the identification of such molecules will improve our understanding of the important role of Vg in facilitating TYLCV movement in the whitefly midgut. In addition, viruses can use multiple receptors to enter cells (7, 42). Prohibitin, phosphatidylserine-mediated virus entry-enhancing receptor, and glycosaminoglycans have all been suggested as chikungunya virus (CHIKV) receptors in mammalian cells (46–48). Two proteins of 60 and 38 kDa were found to be the putative gut receptors for CHIKV in A. aegypti (49). Our most recent study showed that an endocytic receptor complex that is composed of two whitefly proteins, *B. tabaci* CUBN (BtCUBN) and BtAMN, also plays a role in facilitating TYLCV entry into whitefly midgut cells (50). We will go on to determine whether the Vg and BtCUBN/BtAMN pathways function independently or cooperatively in promoting TYLCV entry into midgut cells.

Although the insect *Vg* gene has been previously reported to be expressed in tissues other than the female fat body (28, 34–36), our study demonstrated for the first time that a *Vg* gene is expressed in the midgut of an insect. The physiological function of non-fat-body-produced Vg has been clarified in only a few cases. In *L. striatellus*, Vg protein is synthesized and proteolytically cleaved into the N-terminal small subunit and the C-terminal large subunit in both the fat body and hemocytes. However, the large subunit capable of interacting with RSV is further consumed in the fat body but remains stable in the hemocytes. As a result, only the hemocyte-produced Vg binds to RSV *in vivo* and facilitates the transmission of the virus (36). Our results indicate that the whitefly Vg is also proteolytically cleaved into an N-terminal small subunit and a C-terminal large subunit in both the fat body and midgut after synthesis. However, unlike that in *L. striatellus*, the virus-interacting large subunit exists stably both in the midgut and in the fat body. Thus, Vg protein synthesized in both tissues interacts with TYLCV, where the midgut-produced Vg facilitates the movement of virus crossing the midgut wall to enter hemolymph, while the fat body-produced one promotes the transport of virus from the hemolymph to ovaries (21). Due to the lack of a small-subunit-specific antibody, whether the small subunit of Vg exists in the whitefly midgut and fat body remains uncertain. Our data show that the small subunit can neither interact with TYLCV CP nor bind to the microvillar membrane and is thus likely to be dispensable for virus interaction. However, this subunit contains a signal peptide and is expected to be required for Vg secretion. Here, we addressed the role of midgut-produced Vg in virus recognition and transmission; however, the function of midgut Vg in the absence of virus remains unknown. It has been shown that in addition to supplying developing embryos with amino acids, Vgs are extensively modified, carrying covalently linked carbohydrates, phosphates, and sulfates and noncovalently bound lipids, vitamins, hormones, and metals, thus facilitating the transport of these nutrients from hemolymph to ovaries (29, 51, 52). The insect midgut is a tissue that absorbs the nutrients necessary for insect survival (53). We thus speculate that the midgut-produced Vg may function in mediating the absorption of nutrients from the gut lumen into epithelial cells and finally into the hemolymph in whiteflies.

The Vg protein consists of three Vg-specific functional domains: VitN, DUF1943, and vWD (28). Previous studies have shown that the VitN is required for interacting with VgR in the tilapia Oreochromis aureus and freshwater prawn Macrobrachium rosenbergii (54, 55). In the whitefly midgut, VitN mediates the binding of the large subunit with microvillar membrane. Therefore, the VitN plays a conserved role for receptor recognition in both vertebrates and invertebrates, including insects. The DUF1943 and vWD have been shown to play critical roles in pathogen recognition (56, 57). In Patinopecten yessoensis, recombinant DUF1943 and vWD both can interact with the lipopolysaccharides and lipoteichoic acid expressed on the bacterial cell wall (56). The DUF1943 and vWD of *L. striatellus* Vg are able to interact with RSV CP, whereas the VitN is unable to do so (57). Unexpectedly, TYLCV CP interacts not only with vWD but also with VitN of *B. tabaci* Vg. Comparison of the amino acid sequences of VitN, DUF1943, and vWD of Vg from *B. tabaci* with that from *L. striatellus* revealed an identity of 43.29%, 32.14%, and 26.19%, respectively (see Fig. S6 in the supplemental material). Therefore, the VitN is the most conserved region of Vg, which is likely to be consistent with its conserved function in receptor recognition. In contrast, the DUF1943 and vWD are quite divergent, which may contribute to the interaction of these domains with different pathogens. Future study of the critical amino acids in TYLCV CP and whitefly Vg that are responsible for their bindings would shed light on the evolution of the virus-Vg interactions.

10.1128/mSystems.00581-21.6 FIG S6 Comparison of the amino acid sequences of VitN, DUF1943, and vWD domains from *B. tabaci* with that from *L. striatellus*. The VitN, DUF1943, and vWD domains of MEAM1 whitefly Vg (Bta07851) and *L. striatellus* Vg (AGJ26477.1) were predicted using the Conserved Domain Database (https://www.ncbi.nlm.nih.gov/cdd). Alignments were done by DNAMAN (6.0.3.93). Identical amino acids are shown in black letters on a red background. Download FIG S6, TIF file, 4.4 MB. Copyright © 2021 He et al. 2021 He et al. https://creativecommons.org/licenses/by/4.0/ This content is distributed under the terms of the Creative Commons Attribution 4.0 International license.

In summary, we have demonstrated that the whitefly Vg is synthesized in the midgut, interacts with TYLCV, and facilitates the movement of the virus crossing the midgut wall for efficient transmission. This TYLCV-Vg interaction may be a common molecular mechanism to promote the passage of TYLCV across the midgut wall in different whitefly species, as TYLCV interacts with Vg in the midguts of all three whitefly species examined in this study, and the interaction is required for viral acquisition. However, as the >400 species of begomoviruses are believed to be exclusively transmitted by >40 whitefly species of the *B. tabaci* complex, the specific combinations of virus-vector are numerous; it is yet to be determined how widespread this mechanism involved in virus movement across midgut wall is.

## MATERIALS AND METHODS

### Insects, plants, and virus.

Three cryptic species of the *B. tabaci* whitefly complex, Middle East Asia Minor 1 (MEAM1) (mitochondrial cytochrome oxidase I, GenBank accession no. GQ332577.1), Mediterranean (MED) (mitochondrial cytochrome oxidase I, GenBank accession no. GQ371165), and Asia II 1 (mitochondrial cytochrome oxidase I, GenBank accession no. DQ309077), were reared on cotton plants (Gossypium hirsutum L. cv. Zhemian 1793) in insect-proof cages at 26°C (±1°C) under a photoperiod of 14:10 h (light/dark) and relative humidity of 50% (±10%). The purity of the culture was monitored every three generations by amplifying and sequencing the mitochondrial cytochrome oxidase I gene, which has been used widely to differentiate *B. tabaci* genetic groups (11). Clones of TYLCV isolate SH2 (GenBank accession no. AM282874.1) were agroinoculated into plants of tomato (Solanum lycopersicum L. cv. Hezuo903). Plants were grown in insect-proof greenhouses under controlled temperature at 25 ± 3°C and natural lighting supplemented with artificial lights for 14 h a day from 0600 to 2000 h.

### Tissue collection.

For midgut and fat body isolation, the whiteflies were anesthetized on ice for 5 min and then dissected from the abdomen in prechilled phosphate-buffered saline (PBS) buffer. The midguts and fat bodies were collected separately without contamination from other tissues and placed in PBS buffer. The whiteflies were then dissected from the prothorax for PSG isolation. The midguts and PSGs were washed twice in PBS to remove contaminating viruses or proteins from the hemolymph. For hemolymph collection, each whitefly was dissected from the abdomen in 10 μl prechilled PBS buffer to release the content. Then, all the liquid was collected without contamination from other tissues.

### Preparation of midgut protein and IP-UPLC-MS/MS.

About 6,000 newly emerged whiteflies were allowed to feed on TYLCV-infected tomato plants for 1 week, and then midguts were dissected from these whiteflies and washed twice in PBS before collection. Total protein was extracted from 2,000 midguts using the lysis buffer supplied in the Capturem immunoprecipitation (IP) and coimmunoprecipitation (co-IP) kit (TaKaRa; 635721). The extracted proteins were divided into two equal parts and used for immunoprecipitation (IP), one with a TYLCV coat protein (CP)-specific mouse monoclonal antibody (58) and the other with a mouse preimmune serum (control serum), using the Capturem IP and co-IP kit according to the manufacturer’s instructions. The immunoprecipitates were then digested according to a filter-aided sample preparation (FASP) method (59). The shotgun ultraperformance liquid chromatography-tandem MS (UPLC-MS/MS) procedure and data analysis were performed as previously reported (60). The MS/MS spectra were searched against the peptide database of the MEAM1 species of *B. tabaci* (http://www.whiteflygenomics.org) using the SEQUEST HT search engine configured with a Proteome Discoverer 1.4 workflow (Thermo Fisher Scientific, Bremen, Germany). The search parameters include 10-ppm and 0.8-Da mass tolerances for MS and MS/MS, respectively; trypsin as the proteolytic enzyme with two allowed missed cleavages; oxidation and deamidated as dynamic modifications; and carbamidomethyl as static modification. Further, the peptides were extracted using high peptide confidence. The 1% false-discovery rate (FDR) was calculated using a decoy database by searching the peptide sequence.

### q(RT)-PCR analysis.

Groups of 100 midguts, 100 ovaries, or the fat bodies of 100 whiteflies were used to measure gene expression levels in these tissues, and groups of 20 female or male whiteflies were used for gene expression level determination in whitefly whole bodies. Total RNA was isolated using TRIzol reagent (Ambion; 15596018), and then cDNAs were produced using the PrimeScript RT reagent kit with genomic DNA (gDNA) eraser (TaKaRa; RR047A). For viral DNA load quantification in whitefly whole bodies, total DNA was extracted from groups of 20 female or male whiteflies. Whitefly whole bodies were ground in 40 μl of ice-cold lysis buffer (50 mM Tris-HCl, pH 8.4, 0.45% Tween 20, 0.45% Nonidet P-40, 0.2% gelatin, and 60 mg/liter proteinase K) and were incubated at 65°C for 2 h and then at 100°C for 10 min. The supernatants were kept at −20°C. For viral DNA load quantification in whitefly tissues, one PSG, one ovary, or the hemolymph of one female whitefly was dissected, ground in 10 μl of ice-cold lysis buffer, and then incubated at 65°C for 4 h and finally at 100°C for 10 min. The supernatants were kept at −20°C. q(RT)-PCR was performed using an ABI Prism 7500 Fast real-time PCR system (Applied Biosystems) with SYBR Premix Ex Taq II (TaKaRa; RR820A), and the primers are shown in Table S3 in the supplemental material. For each reaction, 0.8 μl of each primer (10 mM), 6.4 μl of nuclease-free water, and 10 μl of SYBR Premix *Ex Taq* were added, in a total volume of 20 μl. The q(RT)-PCR protocol was 95°C for 30 s, followed by 40 cycles of 95°C for 5 s and 60°C for 30 s. A negative control (nuclease-free water) was included throughout the experiments to detect contamination and to determine the degree of dimer formation. The results (threshold cycle [*C_T_*] values) of the q(RT)-PCR assays were normalized to the expression level of the *B. tabaci β-actin* gene. The relative gene expression level or relative abundance of viral DNA was calculated using the 2^−Δ^*^CT^* method.

10.1128/mSystems.00581-21.9 TABLE S3 Primers used in this study. Download Table S3, PDF file, 0.1 MB. Copyright © 2021 He et al. 2021 He et al. https://creativecommons.org/licenses/by/4.0/ This content is distributed under the terms of the Creative Commons Attribution 4.0 International license.

### Immunofluorescence assay.

Whitefly tissues were dissected freshly and fixed in 4% paraformaldehyde (MultiSciences Biotech; LK-F0001) for 1 h at room temperature and washed in TBST (Tris-buffered saline [TBS] buffer with 0.05% Tween 20) three times. The specimens were then permeabilized using 0.1% Triton X-100 in TBS for 1 h and blocked using TBST-bovine serum albumin (BSA) (TBST with 1% BSA) for 2 h at room temperature, followed by incubation with primary antibody in TBST-BSA overnight at 4°C. The specimens were subsequently incubated with DyLight 488 (MultiSciences Biotech; LK-GAM4882) or DyLight 549 (MultiSciences Biotech; LK-GAR5492) labeled secondary antibody (1:500) in TBST-BSA for 1 h at room temperature after extensive washing with TBST. For intracellular vesicle or actin-based microvillar membrane and visceral muscle staining, the midguts were further incubated with Alexa 647 lectin HPA (Invitrogen; L32454) or DyLight 647 phalloidin (Yeasen; 40762ES75) for 1 h at room temperature. The mouse anti-Vg monoclonal antibody (37) was provided by Gong-Yin Ye, Zhejiang University. The mouse anti-TYLCV CP monoclonal antibody and rabbit anti-TYLCV CP polyclonal antibody were provided by Jian-Xiang Wu, Zhejiang University. The rabbit anti-VgR polyclonal antibody (61) was provided by Xiao-Ping Yu, China Jiliang University. The other antibodies were rabbit anti-GFP monoclonal antibody (Abcam; ab183734) and rabbit anti-Rab5 polyclonal antibody (25). For confocal imaging, samples were mounted in Fluoroshield mounting medium with 4′,6-diamidino-2-phenylindole (DAPI) (Abcam, ab104139) and imaged on a Zeiss LSM 780 confocal microscope (Zeiss, Germany). ImageJ was used to create the fluorescence intensity profiles with default parameters.

### Production of recombinant proteins in *Drosophila* cells.

Sequences encoding the small subunit, large subunit, C-VitN, DUF, and vWD of MEAM1 whitefly Vg (Bta07851) were amplified by reverse transcription-PCR from adult whiteflies, respectively. The primers are shown in Table S3. The PCR products were subcloned into the pAc5.1/V5-His A vector (Invitrogen; V4110-20) with a GFP tag sequence at its 3′ end. All plasmids were sequenced and transfected individually into *Drosophila Schneider* 2 (S2) cells using the Lipofectamine 3000 transfection reagent kit (Invitrogen; L3000008) according to the manufacturer’s instruction. The successful expression of these recombinant proteins was confirmed by appearance of autofluorescence of GFP (green) when visualized under a confocal microscope. Cells transfected with mock vector or vector only cloned with the GFP sequence served as controls. At 72 h after transfection with the relevant vectors, cells were harvested, pelleted at 500 × *g* for 5 min, and then washed twice with PBS. Proteins were extracted using the Minute total protein extraction kit (Invent; SN-002) according to the manufacturer’s instructions.

### Recombinant protein/midgut *in vivo* binding assays.

Midgut binding assays were performed as described previously (39). Briefly, adult whiteflies 2 days posteclosion were fed with a solution containing individual recombinant protein mixed with buffer (PBS) (10% glycerol, 0.01% Chicago Sky Blue, and 5 mg/ml of BSA) for 4 h through a membrane feeding chamber. Then, whiteflies were transferred to another feeding chamber containing a 15% sucrose solution for 6 h to remove unbound proteins in the midgut. Midguts were then dissected from female whiteflies for recombinant protein detection by immunofluorescence staining with a rabbit anti-GFP monoclonal antibody. Whiteflies fed with GFP alone served as controls.

### GST-pulldown and Western blotting assays.

The fragment of TYLCV CP was amplified and cloned into pGEX-6p-1 for fusion with GST. Primers are listed in Table S3. The recombinant protein was expressed in Escherichia coli strain BL21 and purified. The GST-CP was bound to glutathione-Sepharose beads (GE Healthcare; 17-5132-01) for 3 h at 4°C, the mixtures were centrifuged for 5 min at 100 × *g*, and the supernatants were discarded. For mapping of Vg subunits and domains that interact with TYLCV CP, the protein extracts of *Drosophila* S2 cells expressing these proteins were added to the beads, respectively, and incubated for 2 h at 4°C. After being centrifuged and washed five times with PBS, the bead-bound proteins were eluted by boiling in PAGE buffer for 5 min, and then the proteins were separated by 12% SDS-PAGE and detected by anti-His or anti-GFP antibody.

To test the impact of anti-Vg antibody on the interaction between MEAM1 endogenous Vg and TYLCV CP, the nonviruliferous whitefly soluble protein extracts were prepared in cell lysis buffer (20 mM Tris-HCl, pH 7.5, 150 mM NaCl, 1% Triton X-100, 20 mM β-glycerophosphate, 10 mM NaF, 1 mM phenylmethylsulfonyl fluoride [PMSF], 1 mM sodium orthovanadate, 10 mg/ml leupeptin, 2 mg/ml aprotinin, 1 mM EDTA). Mouse anti-Vg monoclonal antibody and the corresponding mouse preimmune sera (control sera) (Beyotime; A7028) as controls were incubated with the whitefly soluble protein extracts for 4 h at 4°C, and then the protein extracts were added to the beads and incubated for 2 h at 4°C. To examine the interaction between MED and Asia II 1 endogenous Vg and TYLCV CP, the soluble protein extracts from these two species were added to the beads and incubated for 2 h at 4°C. The bead-bound proteins were eluted and detected by anti-Vg antibody as described above.

For protein detection in tissues or whole bodies of whiteflies, total protein was isolated from groups of 200 midguts, fat bodies of 200 female whiteflies, or 100 whole whiteflies using the cell lysis buffer. Protein samples were separated by 12% SDS-PAGE and transferred to polyvinylidene difluoride membranes. The membranes were blocked with 5% nonfat milk in phosphate-buffered saline (PBS; Sangon Biotech; SB0627) with 0.1% Tween 20 (BBI Life Sciences; 9005-64-5) and then incubated with the anti-TYLCV CP, anti-Vg, or antiactin (EarthOx; E021020-02) antibodies. After incubation with secondary antibody (MultiSciences Biotech; GAM007), signals were visualized with the ECL Plus detection system (Bio-Rad; 170-5060).

### dsRNA preparation.

Double-stranded RNA (dsRNA) specific to *Vg* (Bta07851) or *VgR* (HM017828.2) of MEAM1, MED *Vg* (GU332722.1), or Asia II 1 *Vg* (GU332721.1) was synthesized using the AmpliScribe T7-Flash transcription kit (Epicentre; ASF3507), following the manufacturer’s instructions. Briefly, the DNA template for dsRNA synthesis was amplified with primers containing the T7 RNA polymerase promoter at both ends (Table S3), and the purified DNA template was then used to generate dsRNA. dsRNA specific to GFP was synthesized as control. Subsequently, the synthesized dsRNA was purified via phenol-chloroform precipitation and resuspended in nuclease-free water, and the concentration of dsRNA was quantified with a NanoDrop 2000 (Thermo Fisher Scientific). Finally, the quality and size of the dsRNAs were further verified via electrophoresis in a 2% agarose gel.

### Gene silencing via oral ingestion of dsRNA.

RNA silencing was performed as previously described (21). Briefly, dsRNAs were diluted into 15% (wt/vol) sucrose solution at the concentration of 300 ng/μl. Approximately 100 adult whiteflies at 1 to 2 DAE were released into each feeding chamber. The tube was incubated in an insect-rearing room for 48 h. Subsequently, RNA was extracted from 20 female or male individuals to examine the gene expression level, and total proteins were extracted from 30 female individuals to examine the Vg protein level. The dsRNA-treated whiteflies were placed to feed on two opposite leaflets of a TYLCV-infected tomato plant for 24 h, using leaf clip cages (18). Then, the whiteflies were collected and used for quantitative assays, immunostaining assays, and virus transmission tests. Each set of experiments was repeated three times.

### Oral ingestion of anti-Vg antibody.

Adult whiteflies at 3 to 4 DAE were collected and fed with anti-Vg antibodies (1:100) or mouse preimmune serum (1:100, control) in 15% (wt/vol) sucrose solution using a membrane feeding device. Approximately 100 adult whiteflies were released into each feeding chamber, and the feeding device was incubated in an insect-rearing room for 48 h. Subsequently, various tissues were dissected from female whiteflies for Vg antibody detection. The antibody-treated whiteflies were placed to feed on two opposite leaflets of a TYLCV-infected tomato plant for 24 h, using leaf clip cages. Then, the whiteflies were collected and used for quantitative assays, immunostaining assays, and virus transmission tests. Each set of experiments was repeated three times.

### Transmission of TYLCV to plants by whiteflies.

In the TYLCV transmission tests, whiteflies after dsRNA feeding were given a 24-h AAP on TYLCV-infected plants and then the whiteflies were inoculated singly to individual uninfected tomato plants. The inoculation was performed on the top second leaf of the plant at the 3- to 4-true-leaf stage (∼3 weeks after sowing) for a 72-h inoculation access period (IAP), using a leaf clip cage. For TYLCV transmission by Vg antibody-treated whiteflies, viruliferous female whiteflies were inoculated singly to individual uninfected tomato plants as described above. The plants were then sprayed with imidacloprid at a concentration of 20 mg/liter to kill all the whitefly adults and eggs and maintained in insect-proof cages at 26°C (±1°C) under a photoperiod of 14:10 h (light/dark) to allow observation of disease symptoms. For each combination, 10 plants per replicate and three replicates were conducted to examine the transmission efficiency.

### Statistical analysis.

Data were presented as mean ± standard error of the mean (SEM) for three independent biological replicates, unless otherwise noted. All analyses were performed using SPSS (version 13) software. Differences between the virus quantities in the hemolymph, PSG, and ovary of whiteflies were analyzed by the nonparametric Mann-Whitney *U* test. The others were assessed with an independent-sample *t* test.

## Supplementary Material

Reviewer comments
