# Supplementary figures and images for: Gut-Expressed Vitellogenin Facilitates the Movement of a Plant Virus across the Midgut Wall in Its Insect Vector
Source: mSystems. 2021 Jun 8;6(3):e00581-21. doi: 10.1128/mSystems.00581-21 (PMC8269243; doi:10.1128/mSystems.00581-21)

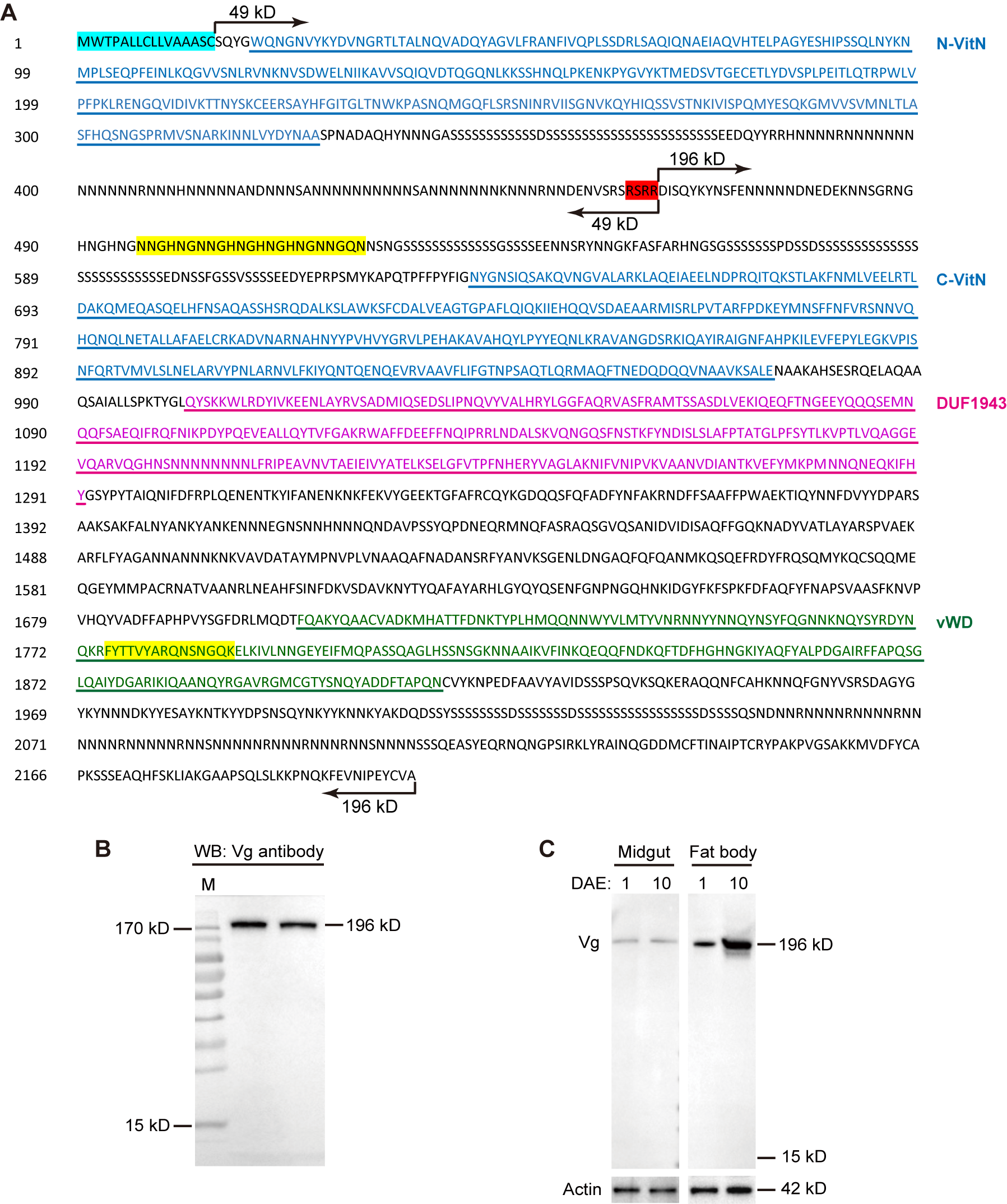

Supplement: FIG S1 [file msystems.00581-21-sf001.tif]

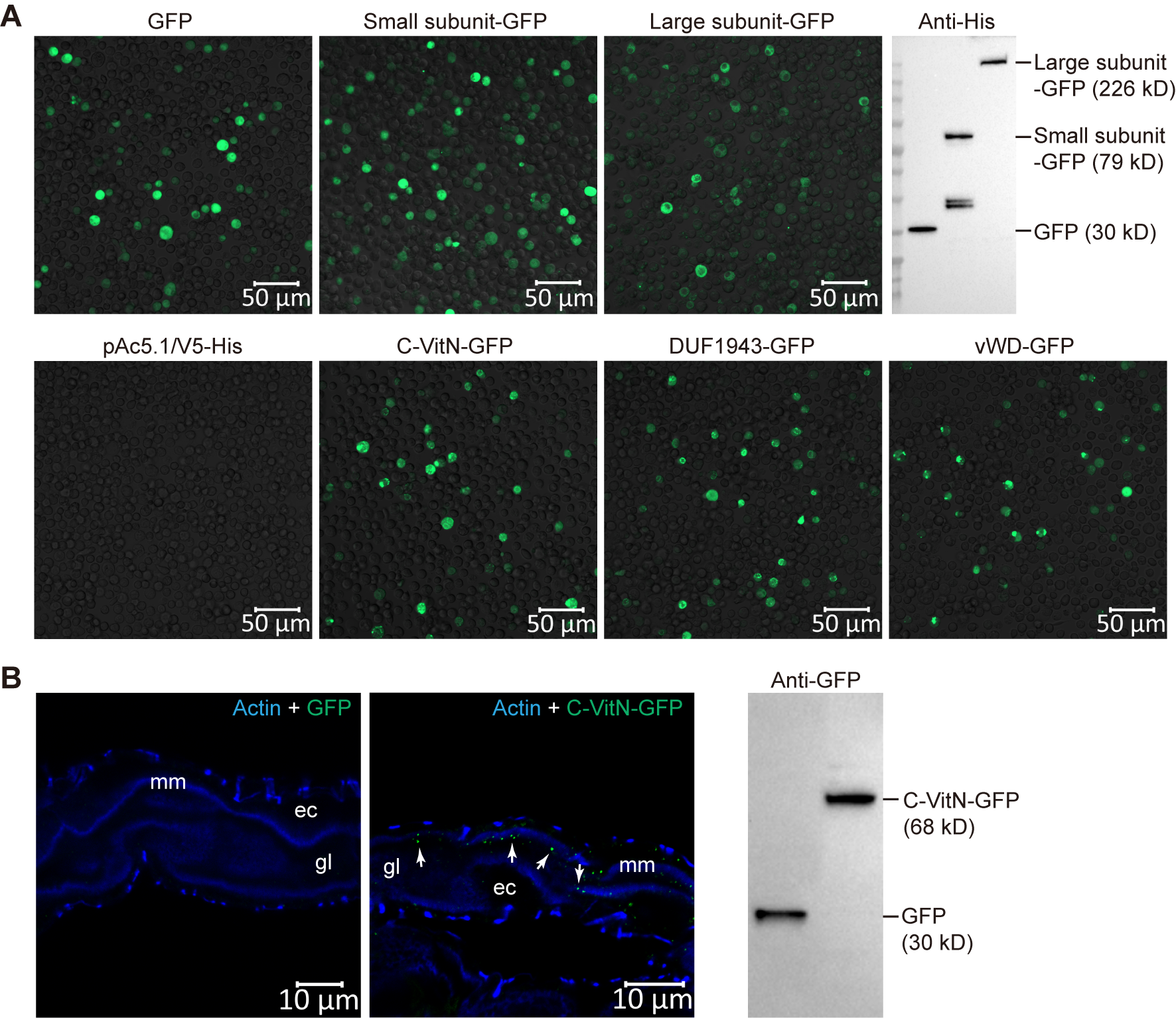

Supplement: FIG S2 [file msystems.00581-21-sf002.tif]

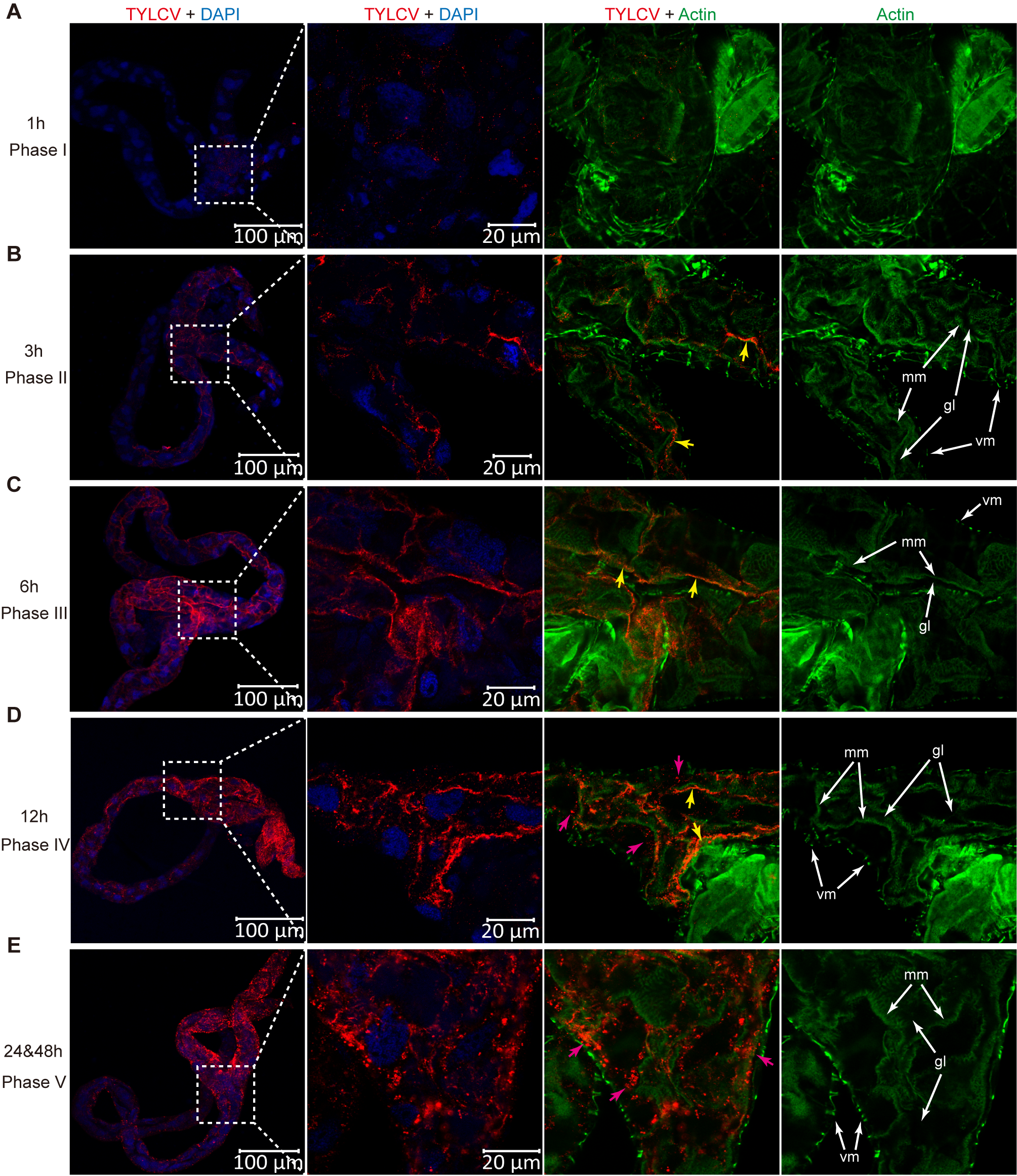

Supplement: FIG S3 [file msystems.00581-21-sf003.tif]

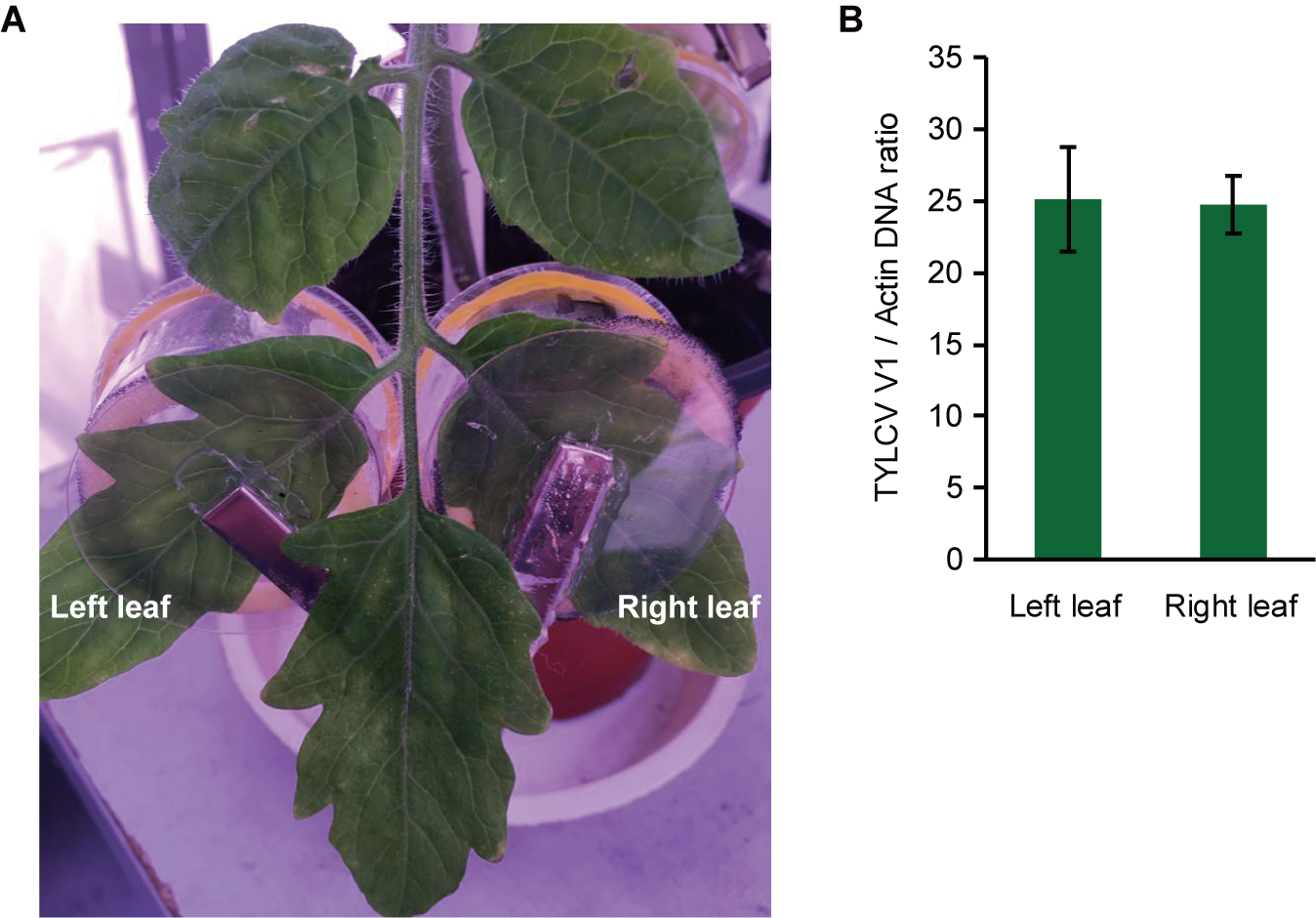

Supplement: FIG S4 [file msystems.00581-21-sf004.tif]

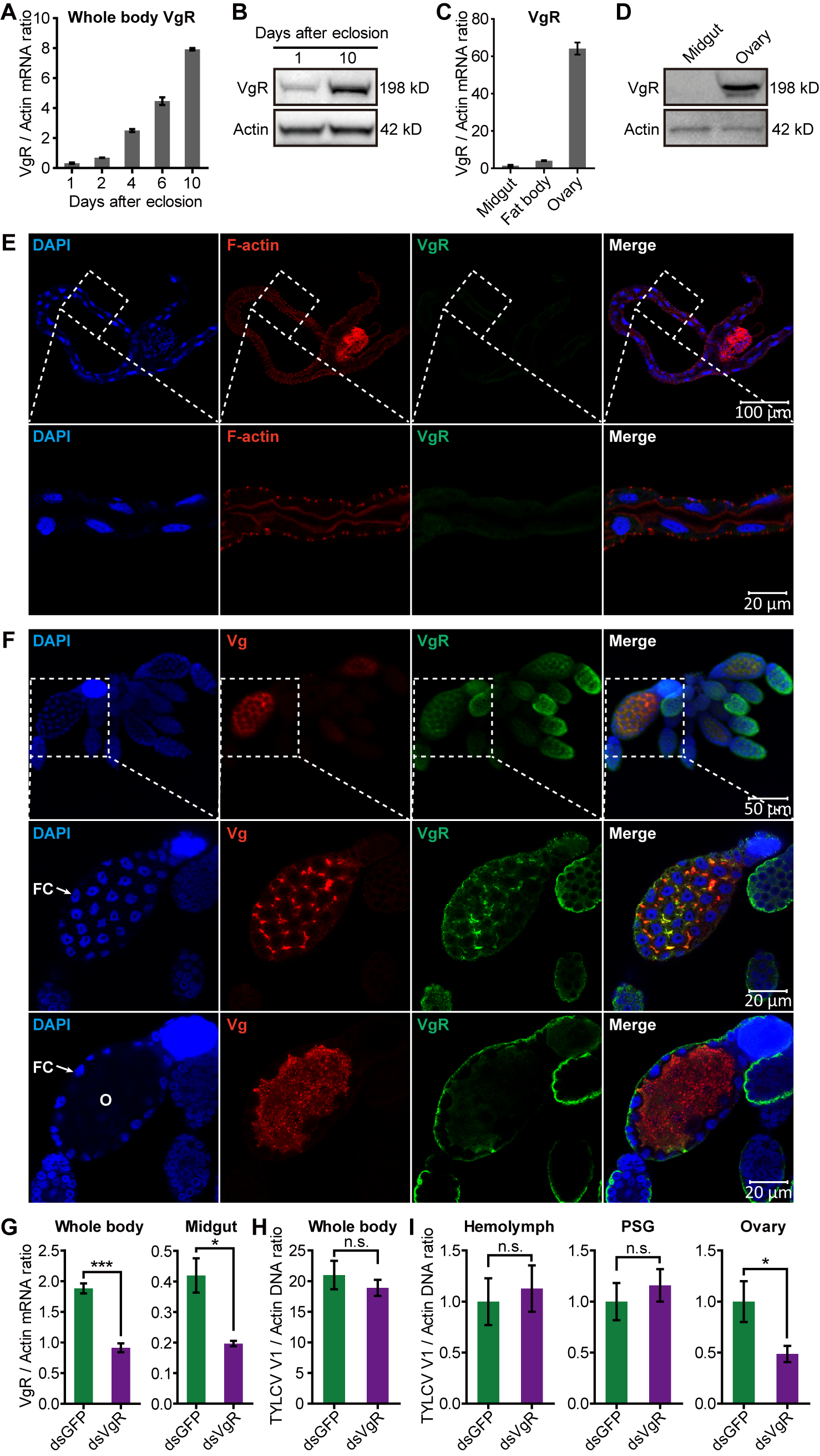

Supplement: FIG S5 [file msystems.00581-21-sf005.tif]

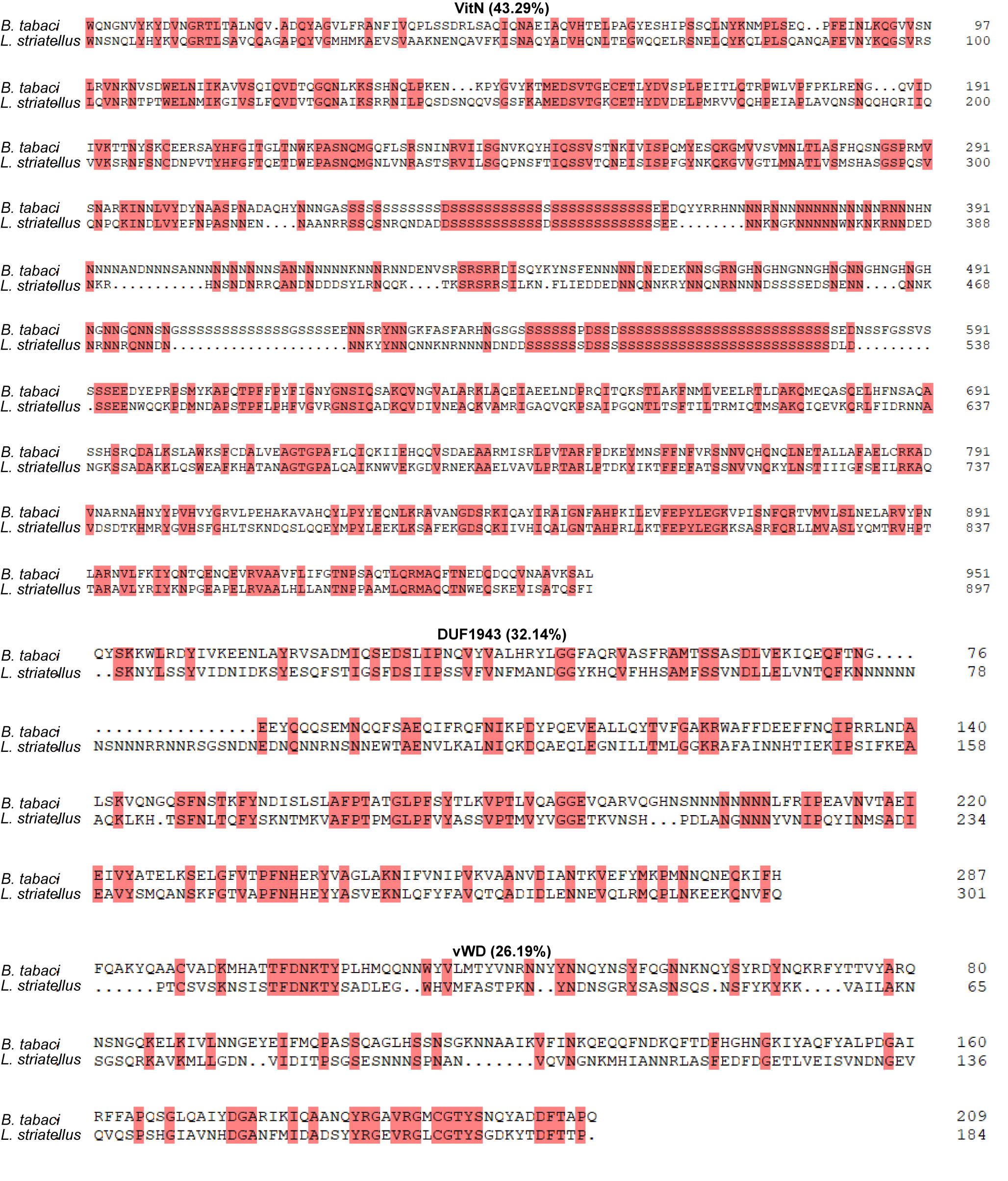

Supplement: FIG S6 [file msystems.00581-21-sf006.tif]
