# Supplementary material for: Gut-Expressed Vitellogenin Facilitates the Movement of a Plant Virus across the Midgut Wall in Its Insect Vector
Source: mSystems. 2021 Jun 8;6(3):e00581-21. doi: 10.1128/mSystems.00581-21 (PMC8269243; doi:10.1128/mSystems.00581-21)
Supplement: TABLE S1 [file msystems.00581-21-st001.pdf]

Table S1. Detection of TYLCV coat protein in the midguts of whiteflies by immunofluorescence microscopy at various times following the first access of whitefly to TYLCV-infected tomato plants.

Hours post first access of whitefly to TYLCV-infected tomato	% Midguts of whitefly with virus in each of the five phases (n=30)*				
	Phase I	Phase II	Phase III	Phase IV	Phase V
0	0	0	0	0	0
1	23	40	7	0	0
3	7	60	17	7	0
6	3	40	44	13	0
12	0	17	13	60	10
24	0	3	7	40	50
48	0	0	0	7	93

*Phase I: TYLCV was only detected in the filter chamber of midgut;
Phase II: TYLCV was detected in the filter chamber, gastric caecum and descending midgut;
Phase III: TYLCV bound to microvilli throughout the whole midgut;
Phase IV: some viral signals were seen in the cytoplasm of epithelial cells;
Phase V: most viral signals were seen in the cytoplasm close to the basal membrane of epithelial cells.
