# Supplementary material for: Gut-Expressed Vitellogenin Facilitates the Movement of a Plant Virus across the Midgut Wall in Its Insect Vector
Source: mSystems. 2021 Jun 8;6(3):e00581-21. doi: 10.1128/mSystems.00581-21 (PMC8269243; doi:10.1128/mSystems.00581-21)
Supplement: TABLE S2 [file msystems.00581-21-st002.pdf]

Table S2. Silencing of Vg or immune-blocking of midgut Vg inhibited the movement of TYLCV crossing the midgut epithelial cells.

**Treatment**		**% Midguts of whitefly with virus in each of the five phases following 24 h acquisition on TYLCV-infected tomato**				
	N.*	**Phase I**	**Phase II**	**Phase III**	**Phase IV**	**Phase V**
dsGFP	60	0	3	10	36	51
dsVg	60	0	7	22	45	26
Control sera	30	0	2	10	33	55
Vg antibody	30	0	6	24	40	30

*The total number of midguts examined for each treatment.
