# Supplementary material for: Gut-Expressed Vitellogenin Facilitates the Movement of a Plant Virus across the Midgut Wall in Its Insect Vector
Source: mSystems. 2021 Jun 8;6(3):e00581-21. doi: 10.1128/mSystems.00581-21 (PMC8269243; doi:10.1128/mSystems.00581-21)
Supplement: TABLE S3 [file msystems.00581-21-st003.pdf]

Table S3. Primers used in this study.

Primer name	Sequence (5'-3')*	Purpose
TYLCV-F	ATACCTGGACACCTAATGGC	TYLCV DNA detection
TYLCV-R	AGTCACGGGCCCTTACA	
qTYLCV-F	GAAGCGACCAGGCGATATAA	q-PCR for TYLCV total DNA
qTYLCV-R	GGAACATCAGGGCTTCGATA	
qMEAM1 Vg-F	ACAAGTCTCCGACGCCGAAG	qRT-PCR for MEAM1 *Vg*
qMEAM1 Vg -R	TTGACATCGGCTTTACGGCA	
qMEAM1 VgR-F	TGGGTCCTGCAAAGCCACTG	qRT-PCR for MEAM1 *VgR*
qMEAM1 VgR-R	TTGGCTCGTGTCTGTGCGTT	
qMED Vg-F	TACGCTGACACTTCACCGCC	qRT-PCR for MED *Vg*
qMED Vg -R	GTCGCTGCGGCTAACGTAGT	
qAsia II 1 Vg-F	CTGCCGTCTACGCTGTCGTT	qRT-PCR for Asia II 1 *Vg*
qAsia II 1 Vg -R	GTCGCTGCGGCTAACGTAGT	
qβ-Actin-F	TCTTCCAGCCATCCTTCTTG	q(RT)-PCR for *β-Actin*
qβ-Actin-R	CGGTGATTTCCTTCTGCATT	
MEAM1 Vg-RNAi-F	T7-ACATCGTCAAGGCCACCAA	MEAM1 *Vg* dsRNA synthesis
MEAM1 Vg-RNAi-R	T7-TAGAGCTGGAACTAGATGAG	
MEAM1 VgR-RNAi-F	T7-GCAGAAGAGGGTGAAGGAC	MEAM1 *VgR* dsRNA synthesis
MEAM1 VgR-RNAi-R	T7-CCAATAGACATGTTTACCATC	
MED Vg-RNAi-F	T7-TAGCAGCGACTCCAGCTCCT	MED *Vg* dsRNA synthesis
MED Vg-RNAi-R	T7-CGGGCTTGGCTGGGTATCTG	
Asia II 1 Vg-RNAi-F	T7-TAGCAGCGACTCCAGCTCCT	Asia II 1 *Vg* dsRNA synthesis

Asia II 1 Vg-RNAi-R	T7-CGGGCTTGGCTGGGTATCTG	
gfp-RNAi-F	T7-CTCGTGACCACCCTGACCTAC	*gfp* dsRNA synthesis
gfp-RNAi-R	T7-GTTCACCTTGATGCCGTTCTT	
GFP-F	ATTTGCGGCCGCATGGTGAGCAAGGGCGAG	Expression of GFP in S2 cells
GFP-R	CCCTCGAGCTTGTACAGCTCGTCCATGC	
Vg small subunit-GFP-F	GGAATTCATGCAATATGGCTGGCAAAACGGAAAC	Expression of Vg small subunit-GFP in S2 cells
Vg small subunit-GFP-R	ATTTGCGGCCGCCAGCGAGAGACGTTCTCGTC	
Vg large subunit-GFP-F	TAGTCCAGTGTGGTGGAATTCATGGATATCTCCCAATACAAATACAACA	Expression of Vg large subunit-GFP in S2 cells
Vg large subunit-GFP-R	CCTTGCTCACCATGAGCGGCCGCCAGGCAACACAGTATTCGGG	
C-VitN-GFP-F	CGGAATTCACTCCTTTCTTCCCCTAC	Expression of C-VitN-GFP in S2 cells
C-VitN-GFP-R	ATTTGCGGCCGCATTCAAGGGCGGATTTGAC	
DUF1943-GFP-F	GGGGTACCCAATACTCCAAGAAATGGC	Expression of DUF1943-GFP in S2 cells
DUF1943-GFP-R	ATTTGCGGCCGCAGGTGTAAGGGTAAGATC	
vWD-GFP-F	CGGAATTCTGCGTTGCTGACAAGATGC	Expression of vWD-GFP in S2 cells
vWD-GFP-R	ATTTGCGGCCGCAACAGTTTTGAGGGGCGGT	
TYLCV CP-GST-F	CGGGATCCATGTCGAAGCGACCAGGCGA	Expression of TYLCV CP in *E. coli*
TYLCV CP-GST-R	CGGAATTCTTAATTTGATATTGAATCAT	

*T7, 5´-TAATACGACTCACTATAGG- 3´
